# Antibiotic perturbation of the murine gut microbiome enhances the adiposity, insulin resistance, and liver disease associated with high-fat diet

**DOI:** 10.1186/s13073-016-0297-9

**Published:** 2016-04-27

**Authors:** Douglas Mahana, Chad M. Trent, Zachary D. Kurtz, Nicholas A. Bokulich, Thomas Battaglia, Jennifer Chung, Christian L. Müller, Huilin Li, Richard A. Bonneau, Martin J. Blaser

**Affiliations:** Department of Microbiology, NYU Langone Medical Center, New York, NY 10016 USA; Department of Medicine, NYU Langone Medical Center, New York, NY 10016 USA; Department of Population Health, NYU Langone Medical Center, New York, NY 10016 USA; Department of Biology, Courant Institute of Mathematical Sciences, New York University, New York, NY 10003 USA; Department of Computer Science, Courant Institute of Mathematical Sciences, New York University, New York, NY 10003 USA; Simons Center for Data Analysis, New York, NY 10010 USA; New York Harbor Veterans Affairs Medical Center, New York, NY USA

## Abstract

**Background:**

Obesity, type 2 diabetes, and non-alcoholic fatty liver disease (NAFLD) are serious health concerns, especially in Western populations. Antibiotic exposure and high-fat diet (HFD) are important and modifiable factors that may contribute to these diseases.

**Methods:**

To investigate the relationship of antibiotic exposure with microbiome perturbations in a murine model of growth promotion, C57BL/6 mice received lifelong sub-therapeutic antibiotic treatment (STAT), or not (control), and were fed HFD starting at 13 weeks. To characterize microbiota changes caused by STAT, the V4 region of the 16S rRNA gene was examined from collected fecal samples and analyzed.

**Results:**

In this model, which included HFD, STAT mice developed increased weight and fat mass compared to controls. Although results in males and females were not identical, insulin resistance and NAFLD were more severe in the STAT mice. Fecal microbiota from STAT mice were distinct from controls. Compared with controls, STAT exposure led to early conserved diet-independent microbiota changes indicative of an immature microbial community. Key taxa were identified as STAT-specific and several were found to be predictive of disease. Inferred network models showed topological shifts concurrent with growth promotion and suggest the presence of keystone species.

**Conclusions:**

These studies form the basis for new models of type 2 diabetes and NAFLD that involve microbiome perturbation.

**Electronic supplementary material:**

The online version of this article (doi:10.1186/s13073-016-0297-9) contains supplementary material, which is available to authorized users.

## Background

Obesity is currently a world-wide epidemic [1] and is linked to metabolic diseases including type 2 diabetes and non-alcoholic fatty liver disease [2]. Obesity heightens the risk for the development of these disorders, yet the relevant mechanisms are not fully understood [2]. However, the microbiota of the gut may be involved in the pathogenesis of obesity, possibly through effects on energy balance, nutrient absorption, inflammatory pathways, and the gut-brain axis [3]; causal interactions among these factors are generally undescribed.

For nearly 70 years, farmers have been giving low doses of antibiotics to livestock to promote their growth [4, 5]. Human and rodent studies have established a strong association between a perturbed microbiome and the development of obesity and related metabolic dysfunction [6–10]. Our prior studies have established models of antibiotic exposures in mice that have led to accelerated growth and to perturbation of host metabolic and inflammatory responses [11–13]. In each of these studies, antibiotic exposure substantially altered the gut microbiota. In a pivotal experiment, colonizing germ-free recipient mice with antibiotic-altered communities of intestinal microbes was sufficient to transfer the adiposity [12]. This established a causal role for what we have termed “microbe-induced obesity” [3]. Adding a high calorie, high-fat diet (HFD) exacerbated the effects of the altered microbiota on both adiposity and hepatic gene expression [11, 12].

In this study, we exposed mice to long-term low dose penicillin (STAT) or not (control), and then changed their diet to HFD to enhance the adiposity phenotype. By studying metabolic and hepatic functions in mature mice (aged >30 weeks), we found that this regimen promoted insulin resistance and hepatic steatosis. Here, we sought to understand the relationship of those phenotypes with metrics related to the gut microbiome. Defining statistical associations between members of the gut microbial community and host phenotypic development in response to perturbation is an essential challenge for inferring mechanism from systems-level data. We describe a novel computational pipeline for estimating the significance of community change upon treatment and for estimating the significance of individual taxa differences between STAT and control.

## Methods

### Animals and exposures

C57BL/6 mice (Jackson Laboratories, Bar Harbor, ME, USA), were allowed to acclimate to our animal facility for 1 week prior to breeding. After 2 weeks, breeding pairs were separated and pregnant dams randomized into control or sub-therapeutic antibiotic treatment (STAT) groups. Penicillin G (6.8 mg/L; STAT) or not (control) was added to drinking water dams at ~ day 14 of gestation, as described [12 13]. Pups were weaned at day of life (dol) 28 and continued receiving the same treatment (STAT or control) throughout the 32-week study. All mice had *ad libitum* access to water and chow (Purina Mills International Diet #5001, 4.07 kcal/g, with 13.5 % kcal from fat). At week 13, all mice were switched onto HFD (4.73 kcal/g, with 45 % kcal from fat; Rodent Diet D12451, Research Diets, New Brunswick NJ, USA). Mice were weighed and fecal pellets were collected regularly throughout the experiment (Additional file 1: Figure S1).

### Body composition

Body composition was measured using dual energy X-ray absorptiometry (DEXA) with a Lunar PIXImus II mouse densitometer (GE Medical Systems, Waukesha, WI, usa) at weeks 4, 8, 12, 20, 24, and 28 with anesthesia by isoflurane inhalation, as described [13].

### Food intake and caloric excretion

At week 21 while receiving HFD, 12 mice (control and STAT males and females; n = 3/group) were individually housed in metabolic cages (Tecniplast, Buguggiate, Italy). The mice were allowed 2 days to acclimate, and then were observed and studied for the next 3 days, with daily weighing of the mice, their food, water, feces, and urine. Caloric intake was calculated as food consumed (g) multiplied by 4.73 kcal/g (Research Diets). Bomb calorimetry was used to quantify calories present in feces. For each mouse, the entire fecal output/24-h period during the 3-day observation period was homogenized, and divided into duplicate (10–20 mg) aliquots, dried overnight at 55 °C with silica gel as a desiccant, and caloric content of the dried aliquots measured in a bomb calorimeter (Semimicro Calorimeter, Thermometer, and Oxygen Bomb; Parr Instrument Company, Moline, IL, USA), using benzoic acid as a standard; mean caloric output was calculated, as described [13].

### Glucose and insulin homeostasis

Intraperitoneal (IP) glucose tolerance tests (IPGTT) and IP insulin tolerance tests (IPITT) were performed during afternoons following 4 h of fasting. For the GTT, mice were injected IP with 1 mg glucose/g body weight in sterile water. Before (time 0), and after (15, 30, 60, and 120 min) the IP injection, blood glucose was measured with an Abbott (Abbott Park, IL, USA) Freestyle Lite glucometer. During the GTT, in seven of the 27 mice tested (3/13 in STAT and 4/14 in control), blood glucose levels between 15 and 60 min were >500 mg/dL. Since this was above the detection limit, such mice were defined as having levels of 500 mg/dL. For the ITT, 0.5 U/g body weight of insulin (Humulin R, Eli Lilly, Indianapolis, IN, USA) was injected IP, and glucose measured as above. In the last hour of the test, 11 of the 27 mice became severely hypoglycemic, unresponsive to noise and physical stimulation. These mice were rescued with an IP glucose solution, removed from further ITT measurements, and returned to their cages with food for observation; rescued mice were defined as having blood glucose levels of 20 mg/dL for the next time point. Homeostatic model assessment of insulin resistance (HOMA-IR) score was calculated by ((glucose mg/dL x insulin mU/L)/405), as described [14]. To determine a normal range for HOMA-IR values in mice, strain/age/diet-matched paired glucose and insulin data were obtained from the literature [15]; since a value of 13.2 separated normal and elevated HOMA-IR scores, we used this to define the upper limit for normal in our study. For grouping purposes, mice were considered to be insulin-resistant when they had ≥2 of the following criteria: HOMA-IR >13.2, impaired glucose tolerance by IPGTT, impaired insulin sensing by IPITT.

### Statistical analysis

We fit a piecewise linear mixed regression model [16] to the weight, fat, lean, GTT, and ITT data to compare the group patterns of change over time during early, middle, post-HFD, and later stages of the experiment. For the weight data, we consider the model with common knots at weeks 5, 13 (when HFD was started), and 22. With this model, we performed the group comparisons of changing group trends over the periods: weeks 3–5, weeks 5–13, weeks 13–22, and week 22–31. Cage information was fitted as a random effect in the model to take account for possible correlations among the mice in the same cage. The MIXED procedures of SAS software (version 9.2; SAS Institute Inc., Cary, NC, USA) were used to perform the tests and calculate the estimates. For fat, lean, GTT and ITT, the models are similar except for using different knots. Both the STAT and Control groups were each composed of five or more cages, across two asynchronous cohorts, in two different mouse facilities. The cage effects- as well as sex- are implicitly accounted for in the multi-level PLS model (see below) since we first subtract the variance between the repeated measures made on the same subject. Therefore, first order effects from factors related to within-subject repeated measures (i.e. cage, sex, aging) are removed. Mathematically, this is equivalent to a linear mixed-effect model but the PLS approach extends to multivariate responses and designs, which accounts for colinearity within the dataset.

### Hormone and cytokine measurements

Serum concentrations of insulin, C-peptide, leptin, ghrelin, IL-6, and TNFα were measured using Multiplex Biomarker Immunoassays for Luminex xMAP technology (Millipore, Billerica, MA, USA; panel MMHMAG-44 k), with reading by Luminex 200 analyzer, as described [13]. These measurements were made using cardiac blood from sacrifice. All mice were fasted for 4 h prior to sacrifice.

### Lipid extraction and measurement

For lipid extraction, based on a modified Folch method [17], ~100 mg of tissue in 500 μL of PBS was homogenized using stainless steel beads for 1 min in a Powerlyzer homogenizer. From each sample, 50 μL was removed for protein analysis (BCA reagent, Thermo Scientific) and 1.5 mL of 2:1 chloroform:methanol added, the solution vortex-mixed, then samples centrifuged for 10 min at 3000 rpm at 4 °C. The organic phase was collected and dried under nitrogen gas. The dried lipid was dissolved in 500 μL of 2 % Triton-X 100 in chloroform, further dried, and then dissolved in 100 μL of phosphate buffered saline (PBS), pH 7.4. Triglyceride and total cholesterol were measured using the Thermo Scientific (Waltham, MA, USA) Infinity assays. Free fatty acids were measured using the Wako NEFA kit (Wako Life Sciences, Richmond, VA, USA). Lipid mass was normalized to protein mass.

### Hepatic gene expression

Tissue was preserved in RNeasy at –80 °C post-sacrifice and RNA was extracted using miRNeasy Mini Kit (Qiagen), essentially as described [18]. In brief, samples were converted into cDNA using SuperScript II Reverse Transcriptase (Invitrogen), and expression determined by real-time quantitative PCR (RT-qPCR), using SYBR Green (Life Technologies) in combination on a 480 LightCycler (Roche). Each well contained 18 uL MasterMix solution (0.0 5uL of 10 uM forward/reverse primers, 10 uL SYBR Green, and 7 uL molecular grade H_2_O). For absolute quantitation, the plasmid standard curve was diluted by tenfold in EB buffer. Primer sequences and annealing temperatures were described [18, 19]. qPCR cycling was optimized to each primer-set to ensure Efficiency >1.90 and Error Rate <0.02. Relative concentrations were calculated using the ΔΔCt method, as described [20], and *p* values calculated using the non-parametric Mann–Whitney *U* test.

### Non-alcoholic fatty liver disease assessment

Liver sections were dissected and fixed in 10 % neutral buffered formalin, then paraffin-embedded. Slides were cut, stained with hematoxylin and eosin (H&E), and Masson’s Trichrome, then scanned at 40× and 200×, and scored for non-alcoholic fatty liver disease (NAFLD), as described [21].

### Microbial community analysis

Total genomic DNA was extracted from frozen fecal samples using the Powersoil DNA Extraction Kit (MoBio, Carlsbad, CA, USA) in 96-well format and the 16S rRNA gene was amplified with barcoded fusion primers, targeting the V4 region, as described [22]. Amplicon pools were sequenced on the 2 × 150 bp Illumina MiSeq platform. The QIIME pipeline [23] was used for quality filtering, demultiplexing, taxonomic assignment, and calculating diversity metrics, as described [12]. Sequencing depth, paired-end joining efficiency, and other quality metrics can be found in Additional file 2: Figure S2. We found no significant differences between males and females in either treatment group by clustering or UniFrac distances (data not shown) or between cages (Additional file 3: Table S1, Adonis test). Since there were no differences and stratification reduces analytical power, the sexes were combined for microbiome analyses. To make the data more interpretable, we edited the OTUs according to their representation amongst the samples. We arrived at 723 OTUs by discarding OTUs that were present in fewer than 10 % of all fecal samples. This was an arbitrary cutoff, used both to reduce the noise of amplicon datasets and to avoid spurious associations when there is a preponderance of zero counts. Linear discriminant analysis effect size (LEfSe) [24] was used to detect significant differences in relative abundance of microbial taxa and predicted KEGG pathways between control and STAT mice. Microbiota-by-age z-scores (MAZ) were calculated as described [25], using the following formulae: Microbial maturity (MM) = predicted microbiota age − median microbiota age of control mice of similar age. MAZ = MM/S.D. of predicted microbiota age of control mice of similar age.

### Supervised classification of disease state

Random forests classification models were built for prediction of disease outcomes (NAFLD/elevated HOMA-IR development) as a function of microbial composition and to predict age as a function of microbial composition, as described [11]. Each model was built by growing 1000 trees per forest and d/3 variables (operational taxonomic units, OTUs) randomly sampled at each split, where d is the total number of OTUs in each model. Model error was calculated using a leave-one-out approach. To avoid bias from uneven sampling efforts, all samples were randomly subsampled at 1000 OTU/sample prior to analysis. Subsampling and analysis was performed in ten independent trials, with results used to calculate mean model error and OTU importance.

### Sparse and compositionally-robust multilevel PLS regression

We developed a novel framework to detect associations between specific taxa in fecal microbiota communities and longitudinally-measured host phenotypes. To overcome the detection of statistically spurious associations, we incorporated: (1) the compositionally robust centered log-ratio (clr) transformation of OTU relative abundance data; (2) variance decomposition for multi-level experimental design; and (3) estimation of a sparse linear model via sparse Partial Least Squares (sPLS) regression for connecting high-dimensional and multi-collinear features (OTUs, taxa) and responses (phenotype measurements). We selected seven host phenotype measurements of interest: Body Fat (Fat), Bone Mineral Content (BMC), Lean Mass (Lean) and Dry Mass Index (DMI) (all measured by DEXA), scale weight (Weight), next closest time point of Weight (Weight + 1), and end-of-life NAFLD scores. OTUs that appeared in fewer than 10 % of samples across the entire dataset were removed, leaving a remaining 723 OTUs of interest across 308 samples. A single pseudo-count was added to the fecal microbiota data, to correct for zero-counts, and then center log-ratio transformed [26]. We then decomposed the resulting OTU features and host response data into the relevant “within-subject” components using the two-factor (antibiotic group and diet switch) variance decomposition, as described [27]. The within-subject component captures experimental perturbation effects by subtracting between-subject variances.

We then applied *L*1-penalized PLS regression to the within-subject data [28–30] and fit a bi-linear model. The number of latent components in the sPLS model is fixed to seven (or to the number of non-zero singular values in the cross-covariance matrix). Model sparsity is controlled via the scalar parameter η that weights the influence of the *L*1 penalty. We used a two-stage approach to find a sparse set of significant OTU-phenotype associations. In the first stage, we used stability approach to regularization selection (StARS [31]); the StARS method has been previously shown to be competitive for graphical model problems of similar complexity and scale [31]. We rebuilt the sPLS model over 50 random subsets of the data over a range of values for η, calculating the fraction of data subsets that included a given OTU in the support (i.e. non-zero model coefficients) at each η. We then computed a summary statistic of overall model stability to select the most stable model that exceeds the variability threshold (0.1 %) [31]. In the second stage, we assessed the statistical significance of individual OTUs in the model by computing empirical *p* values over 2000 bootstrapped PLS models (using the StARS-selected support) *p* values computed for an empirical null model, generated by randomly permuting the data. We used routines from the sPLS and caret libraries in R to developed a custom package (which includes methods for the full pipeline and a similar approach for discriminant analysis [32]) called compPLS (software and supplemental methods are available at http://github.com/zdk123/compPLS).

### Clustering of sPLS scores

We clustered the 308 individual samples based on their seven-dimensional sPLS scores using a finite Gaussian mixture model. An EM algorithm was used to find the optimal number of components, initialized with agglomerative clustering. We used the maximal Bayesian Information Criterion (BIC) to find optimal model type (ellipsoidal, equal orientation mode) and number of clusters (six clusters) (Additional file 4: Figure S3). All clustering computation was done with the mclust package in R [33].

### Estimation of microbial association networks

Each of the six clusters of individuals/experiments corresponds to phenotypically similar samples. For each sample set we learned microbial association networks using the Sparse InversE Covariance estimation for Ecological ASsociation Inference (SPIEC-EASI) framework [34]. Nodes in each network correspond to OTUs and edges correspond to direct signed interactions between OTUs given each environment. We ran SPIEC-EASI in neighborhood selection mode and performed model selection via StARS using a variability threshold of 0.05 %.

### Analysis of microbial association networks

To assess the overall similarity of the six different association networks we enumerated all induced subgraphs (graphlets) composed of up to four nodes in each network and recorded, for each node, the frequency of participation in each subgraph. Following [35] we can use the Spearman correlation matrix among 11 non-redundant subgraph frequencies (orbits) across all nodes as a robust and size independent network summary statistics. Pairwise distances between entire networks are computed by using the Frobenius norm between the correlation matrices (graphlet correlation distance [35]). To achieve a low-dimensional description of network similarities we embedded these distances in Euclidean space using classical MDS.

We also assessed the robustness of the different microbial association networks to random and targeted node removals (“attacks”) [36, 37] using natural connectivity [38] as a general measure of graph stability. Natural connectivity (a variant of the Estrada index of a complex network [39]) is a graph-theoretic measure of global network connectivity that has been shown to be more reliable and sensitive than other stability metrics (such as algebraic connectivity or size of largest component) when evaluating attack robustness of complex networks [38]. We measured how natural connectivity of the microbial network changed when nodes and their associated edges are sequentially removed from the network. We considered three network attack scenarios: (1) uniformly at random node removal; (2) node removal based on betweenness centrality; and (3) node removal based on node degree. Betweenness centrality [40] measures a node’s centrality in a network by calculating the number of shortest paths from all nodes to all others that pass through that particular node. Nodes with high betweenness centrality generally correspond to “bottlenecks” in the network, which play a crucial role in the organization of biological networks [41]. Nodes with high node degree (i.e. number of neighbors) represent “hubs” or keystone species in the network. Sequential removal of nodes based on the ranking of these scores thus represents targeted (worst-case) attacks on network stability. For comparison, the random node removal scenario (averaged over n = 50 repetitions) assesses the baseline robustness of the network.

## Results

### Combining STAT with high-fat diet increases body weight

**Fig. 1:**
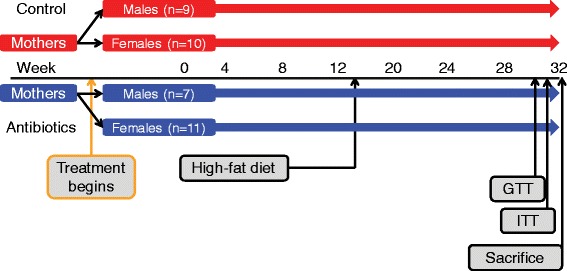
Study design. C57Bl/6 dams were bred, and then randomized to STAT and control groups. Resultant pups continued treatment and were weighed and had fecal samples collected 2–3 times per week until sacrifice at 32 weeks. All mice were switched to a high-fat diet at week 13. A second iteration of this design was performed to increase the number of pups in each group

**Fig. 2:**
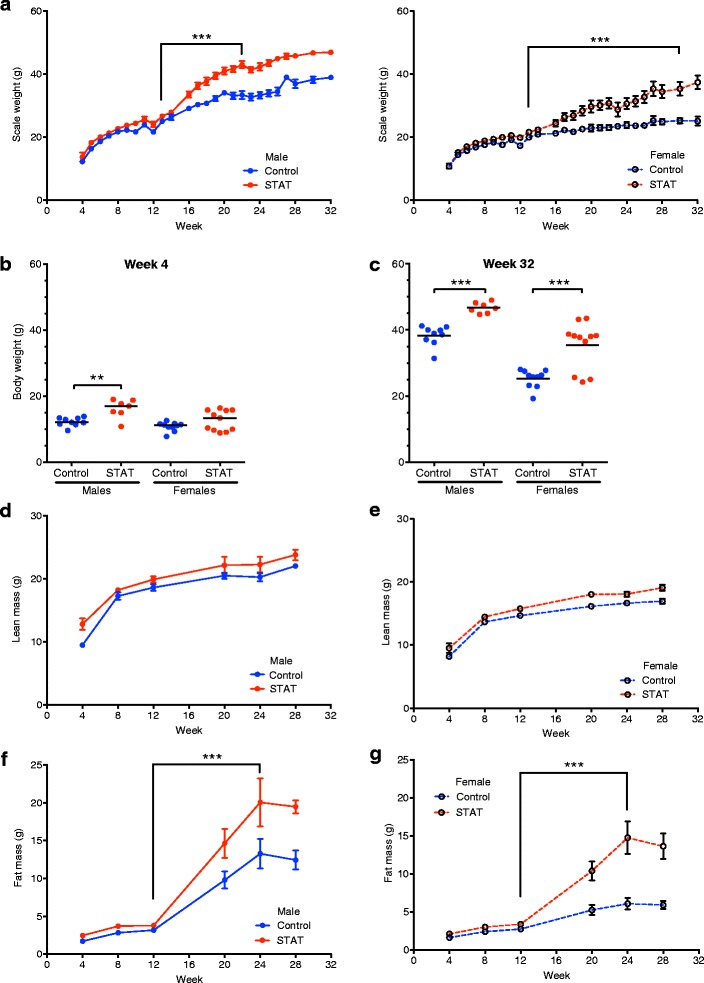
STAT enhances weight gain and adiposity. **a** Scale weight was measured 3–5 times each week beginning at week 4 (day 28) of life. Group data were smoothed to the second order (3-neighbor method). *p* values were calculated using piecewise linear regression to assess rate of growth. **b**, **c** Weight at week 4 (**b**) and sacrifice (week 32; **c**). *p* values reflect ANOVA with Bonferroni’s correction for multiple comparisons. A high-fat diet (45 % kcal from fat) was introduced to all groups at week 13. DEXA was used at 4, 8, 12, 20, 24, and 28 weeks of life and values are shown as Mean ± SD. **d**, **e** Lean mass in male and female mice. **f**, **g** Fat mass in male and female mice. Data in **a**, **d**, **e**, **f**, and **g** are reported as mean ± SEM. *p* values calculated from individual mouse data (Mann–Whitney *U* test). In all panels: *p <0.05; ***p <0.001

We first sought to confirm and extend our prior studies of the effect of STAT on murine development [12, 13], in both males and females (Fig. 1). Analysis of the whole-life growth curves shows that STAT mice were heavier than controls from the very first weights obtained after weaning at week 4 (males only), with differences continuing to the end of the experiment (Fig. 2a–c). Both male and female STAT-exposed mice had increased body weight over time compared to controls, with the major differences occurring after HFD initiation at week 13 (Fig. 2a). After introduction of HFD at week 13, weight gain of STAT mice was greater than in controls (males, 20.0 ± 2.5 g vs. 13.1 ± 3.7 g; *p* <0.001; females, 13.7 ± 5.8 g vs. 5.1 ± 2.4 g; *p* <0.001), showing that the antibiotic exposure potentiated the effects of the HFD. At 32 weeks, both STAT males and females remained significantly larger than controls (Fig. 2c). These studies confirm our prior findings of enhanced growth of mice in the STAT model [12, 13], with acceleration of the growth differences in the presence of HFD.

### STAT with a high-fat diet increases body fat

Beginning at weaning, body composition of all mice was measured by DEXA. Although STAT mice tended to have slightly higher lean mass (Fig. 2d, e), the significant weight differences observed largely reflected fat mass (Fig. 2f, g), which were enhanced by HFD in both sexes. Measurements of bone composition (mineral density, mineral content, and area) were not significantly different in relation to sex, treatment, or diet throughout the experiment (Additional file 1: Figure S1, Panels E, F, and G respectively). Taken together, these data indicate that STAT led to weight gain predominantly in fat mass, beginning early in life, exacerbated by HFD, with little or no effect on lean mass or on bone development, under the conditions studied.

### STAT does not markedly perturb host energy balance

To determine whether STAT was altering food intake or energy harvest, 21-week-old mice were studied in metabolic cages. For individually housed control and STAT male and female mice, we measured food and water intake and waste production for 5 days. Food intake in STAT males was not different compared to controls, but STAT females consumed fewer total calories daily than control females (Additional file 1: Figure S1A). Fecal calorie content (per gram) measured using bomb calorimetry did not vary by sex or exposure group (Additional file 1: Figure S1B). Neither net calories (Additional file 1: Figure S1C; calories IN minus OUT), nor the proportion of calories retained (Additional file 1: Figure S1D; IN minus OUT/IN) was altered by STAT exposure. These data provide evidence that STAT-related adiposity did not result from either increased appetite or enhanced energy harvest.

### STAT affects glucose and insulin homeostasis

**Fig. 3:**
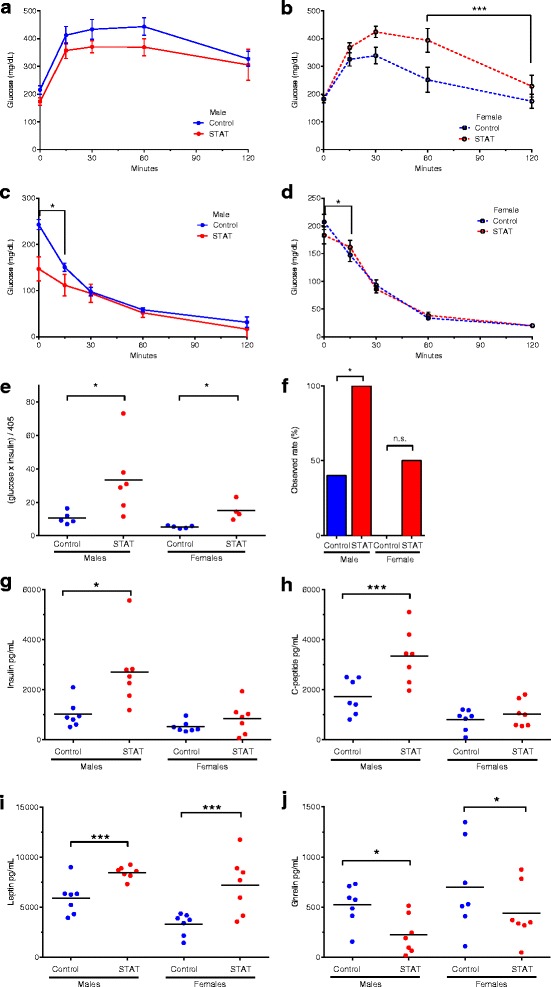
STAT disrupts glucose homeostasis, promoting insulin resistance. For glucose and insulin tolerance testing of 30-week-old male and female mice, six mice from each group were challenged with 5 g/kg dextrose (IPGTT), or with 0.5 U/kg human insulin (IPITT) by intraperitoneal injection. Blood glucose was measured by glucometer at 0, 15, 30, 60, and 120 min post-injection. *p* values reflect differences in rates of change comparing STAT and control. **a**, **b** Glucose tolerance. **c**, **d** Insulin resistance. **e** HOMA-IR was computed as ((Glucose mg/dL) × (Insulin mU/L)/405), as described [1] with values measured at fasting (time 0). *p* values determined by Kruskal–Wallis test (*p <0.05). **f** Observed mice with elevated HOMA-IR (>13.2). *p* values calculated by Fisher’s exact test (*p <0.05). Serum was collected at 32 weeks for analysis by MILLIPLEX® MAP Magnetic Bead Panel. **g** Insulin, **h** C-peptide, **i** leptin, and **j** ghrelin. Each point is the mean of duplicate tests. Data in **a**, **b**, **c**, and **d** are reported as mean ± SEM. *p* values determined by Kruskal–Wallis test (in all panels: *p <0.05; **p <0.01; ***p <0.001)

Based on the increased weight and adiposity phenotypes, we hypothesized that STAT would increase the incidence and severity of metabolic diseases, including type 2 diabetes (T2DM) and NAFLD. To address this hypothesis, we conducted several studies in STAT and control mice in the weeks prior to sacrifice, including tests of glucose and insulin tolerance. While there was no significant difference in recovery of glucose levels in the STAT and control mice, both groups had markedly impaired glucose tolerance and incomplete recovery (Fig. 3a, b); the obese mice in this study were highly glucose intolerant, regardless of STAT exposure.

In insulin tolerance tests, there was significant insulin resistance in both STAT males and females compared to controls (Fig. 3c, d), in the earliest time period after the insulin provocation. Because many of the control animals experienced severe hypoglycemic shock and had to be withdrawn from the test prior to 120 min, we lacked sufficient power for assessment across the usual course of the ITT. At the relatively high level of insulin used, the STAT mice were less sensitive to hypoglycemia than were the controls, due to their relative insulin insensitivity (resistance).

To further quantify the metabolic impact of STAT, we calculated the HOMA-IR index [14]. By this index, based on fasting glucose and insulin values, STAT was found to significantly increase insulin resistance (Fig. 3e) in both males and females. Alternatively, using a pre-defined threshold for elevated HOMA-IR scores, STAT males had a significantly higher incidence of elevated HOMA-IR (Fig. 3f; *p* <0.05) compared to controls. Although not statistically significant, only STAT females, and not control females, had elevated HOMA-IR scores (Fig. 3f). These results, consistent with the ITT results, point to substantial alterations in glucose regulation in the STAT/HFD model.

### STAT affects metabolic hormones and inflammatory markers

Based on the altered glucose homeostasis observed in STAT mice, we measured six other hormones and inflammatory markers involved in metabolism, which we hypothesized would be differentially affected by the STAT exposure. As expected from the IPGTT and IPITT, fasting serum insulin (*p* <0.05; Fig. 3g) and C-peptide (*p* <0.001; Fig. 3h) were significantly elevated in STAT males, although not significantly affected in females. Concordant with the increased adiposity, serum leptin was increased in both STAT males and females (*p* <0.001 in both; Fig. 3i). In contrast, serum ghrelin levels were significantly lower in STAT males and females compared to control mice (*p* <0.05 in both; Fig. 3j). Since metabolic and sex differences may be related to levels of the pro-inflammatory cytokines-TNFα and IL-6, respectively [42], we examined these in the context of the experiment. Circulating IL-6 was significantly elevated in females (*p* <0.05; Additional file 5: Figure S4A) but not in males, and circulating TNFα (Additional file 5: Figure S4B) was not significantly elevated in either sex. These data reflect the enhanced obesity in mice exposed to both STAT and HFD, and provide further definition of the sex differences observed.

### STAT affects hepatic steatosis

**Fig. 4:**
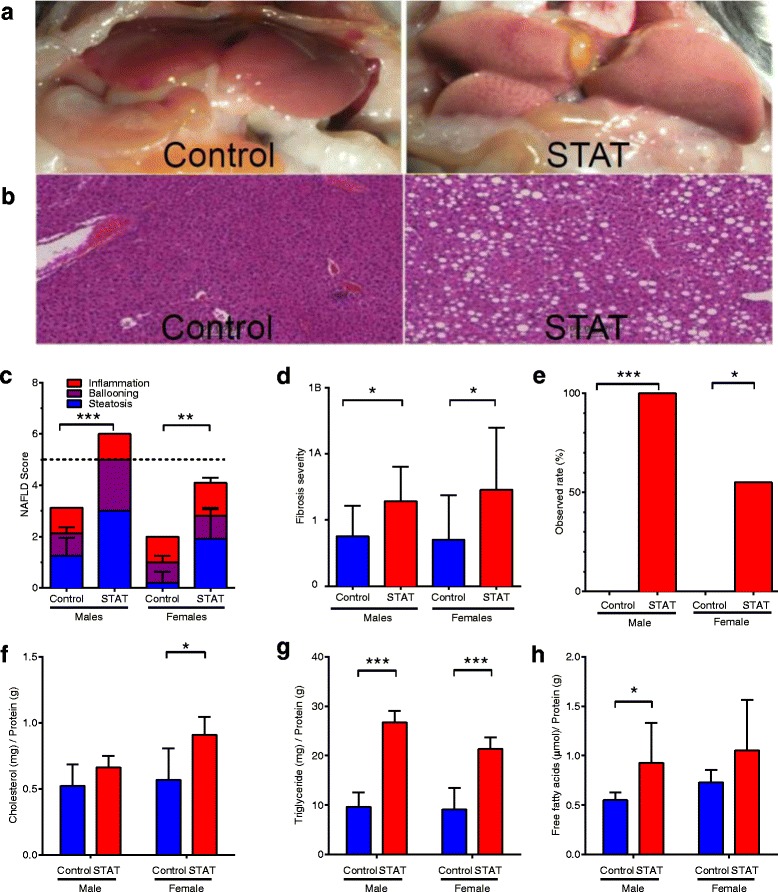
STAT promotes NAFLD through hepatic lipid accumulation. **a**, **b** Ex vivo images and H&E stained slides (magnification × 40), showing the scope of liver pathology. NAS score and fibrosis were determined by standardized histological scoring methods [2] with blinded readers averaging the results of ten fields per mouse for each criterion tested. **c** NAS score by group. The dashed line denotes the diagnostic threshold (>5) for NAFLD. **d** Fibrosis extent and severity scored from trichrome-stained sections. **e** Observed percent of mice with diagnostic NAFLD scores (>5; *p* value by Fisher’s exact test). *p* values were calculated by Kruskal–Wallis test, unless noted. Lipids were extracted from frozen livers, quantified, and normalized to protein. **f** Cholesterol, **g** triglycerides, and **h** free fatty acids. Data in **c**, **d**, **f**, **g**, and **h** are reported as mean ± SEM. *p* values were calculated by paired *t*-test. In all panels: *p <0.05; **p <0.01; ***p <0.001

Upon sacrifice, fatty infiltrates in the liver were visible in 13 of 37 mice (Fig. 4a; 10/18 in STAT, 3/19 in control; *p* = 0.017). Based on these observations, we performed microscopic examination of the liver, grading histology using the NAFLD Activity Score (NAS) [21] (Fig. 4b). Scores for all STAT males were above the diagnostic level for NAFLD with values significantly higher than for controls (*p* <0.001) (Fig. 4c). Although hepatic injury was not as advanced in STAT females, values were significantly higher than in controls (*p* <0.01) (Fig. 4c). Fibrosis (Fig. 4d), evaluated using the same scoring system, was more severe and extensive in STAT than controls (*p* <0.05) in both males and females. Significantly more STAT mice had scores diagnostic for NAFLD (score >5, with fibrosis) compared to controls (Fig. 4e; *p* <0.001 males; *p* <0.01 females). These findings indicate that the combination of STAT and HFD increased the incidence and severity of NAFLD-like histologic lesions compared to HFD alone.

### STAT alters hepatic lipid storage and metabolism

Based on the hepatic histology at week 32, we quantified the hepatic lipid content. Total cholesterol was increased in STAT mice to a greater extent than in controls (*p* <0.05) (Fig. 4f) in females, but not males. In both sexes, STAT livers had nearly twice the triglyceride concentration of controls (*p* <0.001 for each comparison) (Fig. 4g). Free fatty acids were elevated in STAT compared to controls (*p* <0.05) (Fig. 4h) in males, but not females, representing another instance of sex differences in responses to STAT.

Next, we assessed expression of several genes relevant to steatosis. *Cd36* and *Vldlr* expression were increased in STAT to a greater extent than in control (Additional file 5: Figure S4C; *p* <0.05 in both), consistent with the increased lipid infiltration of the liver. However, genes involved in fatty acid metabolism, lipid droplet formation, fatty acid oxidation, and related transcription factors did not differ. When the data were analyzed by outcome rather than treatment group, expression of the cholesterol efflux regulator *Abca1* was lower in mice that had more insulin resistance (Additional file 5: Figure S4D; *p* <0.05). Conversely, *Cd36* was borderline elevated in mice that had increased insulin resistance (Additional file 5: Figure S4E; *p* = 0.055) and in those that had met criteria for NAFLD (Additional file 5: Figure S4F; *p* = 0.055).

### STAT effects on phylogenetic diversity of the intestinal microbiota

**Fig. 5:**
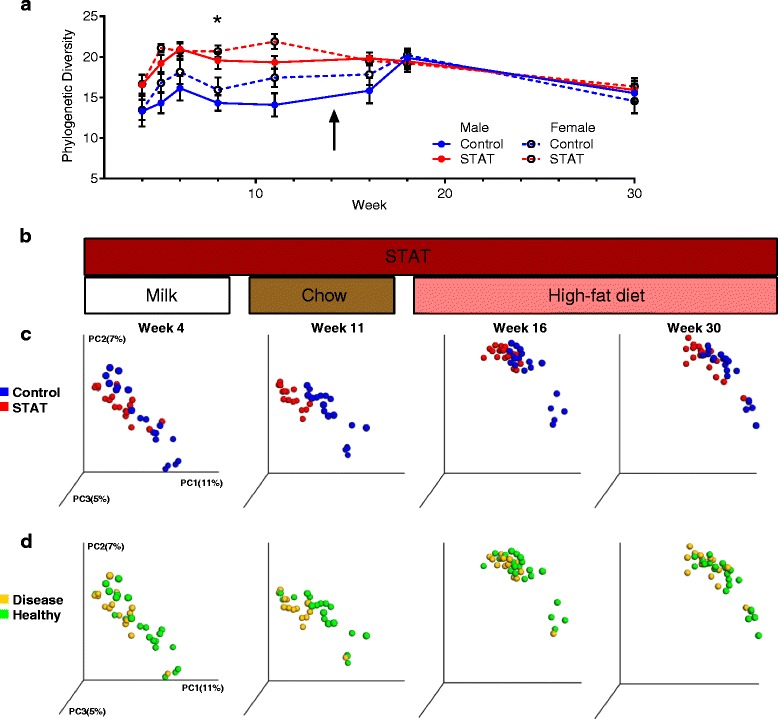
STAT alters microbial communities. **a** α-diversity of all samples over time, rarefied to a depth of 1014. Only differences observed at week 8 were significant (*p* <0.05). **b** STAT exposure and diet corresponding to the PCoA at weeks 4, 11, 16, and 30. **c**–**d** PCoAs of beta diversity at weeks 4, 11, 16, and 30. **c** Control vs. STAT, **d** healthy vs. disease outcome. *p* values calculated by Kruskal–Wallis and AUC analysis (*p <0.05; **p <0.01; ***p <0.001). Adonis testing also indicated significant differences (*p* <0.0005) between the UniFrac distances for the diet:treatment and diet:disease features, when accounting for the repeated measures design (Additional file 3: Table S1)

To assess the relationship of the phenotypic changes to gut microbial composition, we first addressed parameters of community ecology, beginning with markers of community richness. Although α-diversity values were generally higher for the STAT mice compared to controls early in the experiment, the only significant difference (*p* <0.05, Mann–Whitney *U* test) was at week 8 (Fig. 5a).

### Microbiota community structures are distinct between groups

To assess the microbial community structure determined by unweighted UniFrac analysis of the studied samples, we visualized selected time points in relation to dietary transitions by principal coordinates analysis (PCoA) (Fig. 5c). The weeks shown represent the last sample before weaning onto normal chow (week 4), prior to the transition from normal chow to HFD (week 11), shortly after the transition (week 16), and study end (week 30), respectively.

Three female mice received STAT but did not show changes in any phenotypic changes specific to the STAT exposure (see Fig. 2c; these mice were termed female non-responders (FnR)). Based on UniFrac distances, at week 4, community structure of two of the FnR mice were STAT-like, while the third was control-like (*p* >0.05), but by week 11, all three FnR communities were indistinguishable from those in other STAT-exposed female mice, continuing through week 30 (*p* <0.05, compared with controls at each week; data not shown). These findings suggest that microbiota differences linked to differential outcomes in the FnR mice may have occurred prior to week 11.

When samples were grouped by treatment, the UniFrac distances were significantly different between intra- and inter-group measurements at each week (Additional file 6: Figure S5; *p* <0.005 for all weeks), indicating that the community structures of the control and STAT groups were distinct. When specimens were grouped by clinical phenotype (NAFLD, insulin resistance) or by not showing the pre-defined disease definitions (healthy), there were distinct differences at weeks 4, 11, and 30 (Additional file 6: Figure S5; *p* <0.005), but not at week 16. These results provide evidence that before HFD initiation, the intestinal microbial communities in mice that developed disease were distinct from those that did not. Although the addition of HFD diminished this distinction, the communities again were separate, long after the transition (week 30).

### Differentiating taxa

**Fig. 6:**
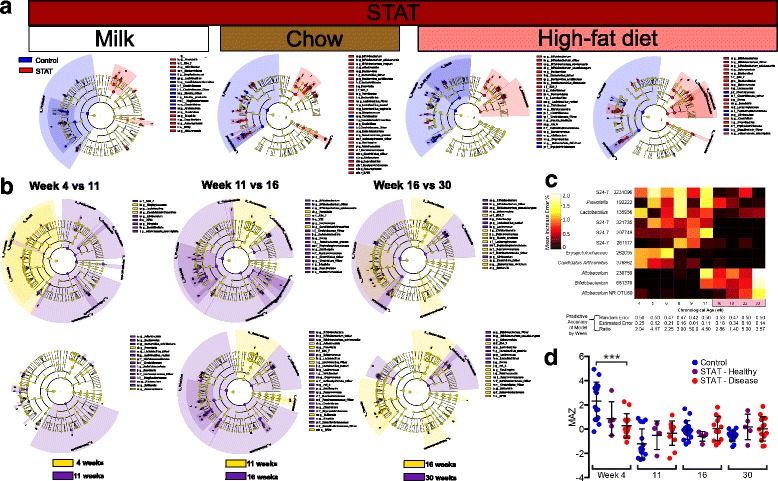
Differential microbial features between STAT and control. **a** LEfSe cladograms showing discriminant taxa between control and STAT at weeks 4, 11, 16, and 30, respectively, with corresponding diet. All identified taxa were significantly altered by Kruskal–Wallis test (*p* <0.05) and had at least twofold increase by LDA. **b** Inter-week comparisons in control (*upper*) or STAT (*lower*). The week 4 to 11 comparison shows changes across weaning, the week 11 to 16 comparison shows changes from the introduction of HFD, and the week 16 to 30 comparison shows changes with increasing age. **c** A Random Forest classification model was built to predict disease outcome (class) based on bacterial OTU relative abundance (features) for each week of life. *Heat map* indicates the importance of each OTU (as mean increase error %) to the disease prediction models at each stage of life. The mean increase error for each OTU indicates the incremental decrease in prediction accuracy if that OTU is removed from the model. *Highlighted time points* show HFD. The table lists the predictive accuracy of the model by week. **d** Average microbiota-by-age z-score (MAZ) over time; z-score = 0 indicates appropriate maturation over time; higher or lower z-scores indicate accelerated or delayed microbiota development, respectively. ***p <0.001 relative to Control, one-way ANOVA with Fisher’s LSD adjusted for false-discovery rate

On the day of weaning (week 4), control mice were enriched in Firmicutes and Candidatus Arthromitus (“Savagella”) (SFB), while STAT mice were enriched in Bifidobacterium, S24-7, and Prevotella, as determined by LEfSe [24]. While some individual taxa differed, that pattern was unchanged while the mice were receiving normal chow (week 11). When the mice were receiving HFD (week 16), the patterns continued similarly, except that SFB and Prevotella differences disappeared, and Allobaculum and Actinobacteria, enrichment was seen in control and STAT mice, respectively (Fig. 6a).

In controls, the week 4 to 11 transition showed a shift from Firmicutes-dominance, whereas the STAT transition was accompanied by a bloom in Proteobacteria (Fig. 6b). The transition between weeks 11 and 16 differed from the earlier transition, with the selective power of HFD having similar effects on control and STAT mice. The further transitions between weeks 16 and 30 similarly affected control and STAT communities (Fig. 6b). Thus, the effects of HFD on individual taxa appear to overwhelm the continuing effects of STAT.

Based on the LEfSe results, we sought to determine if any taxa could predict whether a host would develop metabolic disease (defined as insulin resistance or NAFLD). To accomplish this, a Random Forest classification model was built to predict disease outcome (class) based on bacterial OTU relative abundances (features) for each week of life. Of particular interest was the observation that for early weeks (prior to week 6); six OTUs were predictive (Prevotella, Lactobacillus, Erysipelotrichaceae, SFB, and two different S24-7 OTUs). The model had substantially (more than twofold) better than random predictive power at nearly all time points (Fig. 6c).

To understand the developmental differences in microbial communities, we calculated microbiota-by-age z-scores (MAZ) [11, 25] to compare the communities observed in control and STAT that did not develop disease, with the STAT mice that did (Fig. 6d). Intestinal microbiota follow reproducible patterns of community succession during early life, allowing “microbiota age” to be used as a benchmark of normal intestinal development, as described in studies of humans [25]. In this model, a maturity difference from control indicates either accelerated or delayed development of an age-appropriate microbial community. At week 4, samples from the STAT mice that would later develop insulin resistance or NAFLD had significantly lower MAZ scores than controls (*p* <0.001), but differences were lost at weeks 11, 16, and 30. These data provide evidence that STAT can delay the normal development of the early-life microbiome, and that this delay is associated with elevated risk for metabolic diseases in later life.

### Associations between host phenotypes and microbial taxa

**Fig. 7:**
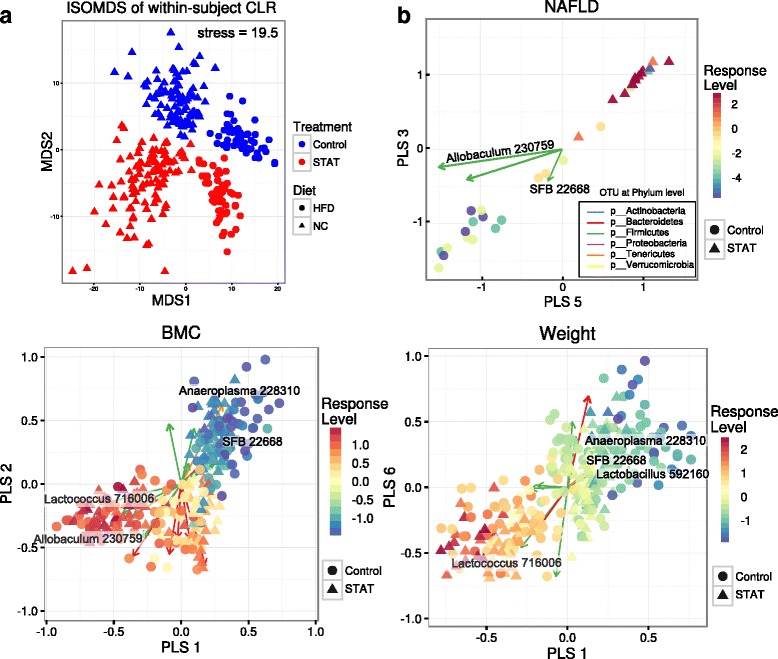
Fecal microbial compositions are associated with host body phenotypes and disease indications. **a** Isometric multidimensional scaling (MDS) of Euclidean distances between clr-transformed OTU compositions, with within-subject variances extracted. The first two MDS components are shown, with Control vs. STAT and NC vs. HFD (point color, shape) explicitly modeled in this approach. This was done by evaluating between-subject variances within each respective group and subtracting from the full dataset. **b** Within-subject response-selected OTUs are shown as biplots. For each phenotype of interest (NAFLD, BMC, or Weight), the relevant two-component (out of seven possible latent components) subspaces from the sPLS model are shown. Taxa are filtered for statistical significance (α = 10^–2^) and key taxa are highlighted for biological significance. “Response Level” indicates the centered and scaled within-subject variances of the relevant measurement

We applied multi-level, sparse PLS models to fecal microbiota data to assess linear relationships between OTUs and seven host phenotypes (Fat, Lean, BMC, DMI, Weight, Weight + 1, and NAFLD). We verified the efficacy of a multi-level linear model by visualizing the within-subject portion of the clr-transformed data. ISOMDS indicates clear separation between subjects of differing groups (Fig. 7a compared to Additional file 4: Figure S3A). We also computed biplots for the sPLS model (Fig. 7b and Additional file 7: Figure S6B), with sample scores colored by (scaled and centered) response variable and significant OTUs, represented by a loading vector colored by phylum.

Overall, 29 taxa (about 4 % of the total) were selected by the fully specified sPLS model, and three additional OTUs (two Clostridiaceae, and Odoribacter) were found to not be significant at α = 10^–2^. However, we found a large number of significant associations between taxa and body composition phenotypes (Additional file 8: Table S2). With the exception of two S24-7 families, all other Bacteroidetes OTU abundance levels were positively associated with body mass phenotypes, while Firmicutes associations were mixed.

More specifically, we found that Lactobacillus (n = 2) OTUs to be significantly associated with Lean, BMC, DMI, and Weight and one other Lactobacillus directly associated with Fat, DMI, Weight, and Weight + 1. This is consistent with prior findings that *Lactobacillus reuteri* reduces abdominal fat and age-associated weight gain [43]. *Turicibacter* genera (n = 2) were found to be negatively associated with DMI and Fat, but were not significantly associated with other body composition measurements, consistent with prior studies of low-dose antibiotic exposure [12] and HFD feeding [44] in mice. A single Anaeroplasma genus was negatively associated with BMI, but not NAFLD, which is consistent with HFD administration in C57BL/6 J mice [45] and abundance enrichment in low-weight rabbits [46].

Notably, we found a negative association between an Allobaculum OTU and NAFLD, accompanied by significant positive associations to other body composition measurements. Our findings are consistent with the previous observations that Allobaculum has been directly correlated with adiposity after switch to a HFD [12], yet negatively correlated with the development of the metabolic syndrome and total cholesterol levels [47, 48]. Finally, we also find Candidatus Arthromitus (SFB, n = 3 OTUs) to be negatively associated with body composition phenotypes, primarily Weight, BMC and Lean (consistent with elevated levels of SFB in control vs. STAT mice reported in [12]) with one particular SFB OTU predicted to have additional associations with NAFLD, Fat, and Weight + 1.

### Microbial network topology corresponds to host physiology

**Fig. 8:**
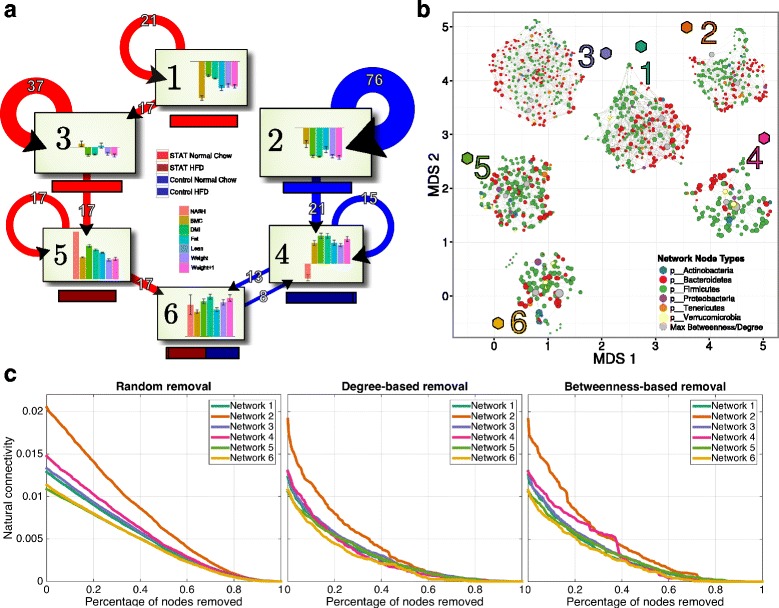
Network properties recapitulate physiology. **a** For each of the six clusters, which were defined from clustering scores in the multilevel sPLS model-fitted subspace, we show the treatment group identity (STAT/Control and NC/HFD, *colored horizontal bars*) and average physiological responses (*vertical bar plots*). Since each response is scaled and centered, the axes represent the mean response over the whole population at each time point. The state-change diagrams represent real-time transitions for the community in an individual mouse moving into a new cluster. For greater clarity, we removed transitions representing fewer than six mouse cluster changes. Clusters 1 and 3 are predominantly obtained from communities in STAT mice early-in-life, and Cluster 2 represents the early-in-life communities in control mice. The switch from NC to HFD corresponds to transitions from Cluster 3 to 5 and from Cluster 2 to 4. Transitions to Cluster 6 primarily include samples from week 30 STAT mice and week 18 and 30 Control mice. The *circular arrows* shown indicate those communities in mice that do not change clusters **b** We inferred networks using SPIEC-EASI [34] over the set of samples defined by each cluster. To compare graphs, we include a two-dimensional embedding of graphlet correlation distances (using isometric MDS, with the network positions shown as colored hexagons). These show that based on summarized local network topologies, closeness networks reflect cluster identity. The networks are shown in force directed layouts (overlaid on the ISOMDS, near their respective position in the embedding) and nodes are colored at the Phylum level, except for the two nodes with the highest betweenness (shown in *gray*, see also Additional file 6: Figure S6). **c** We used natural connectivity to assess the robustness of microbial ecological interaction networks to sequential node removals. The order of node removals was either random or ordered by degree or betweenness centrality. Natural connectivity is shown as a function of the relative size of the network

We next sought to develop a network model that would permit insights about microbial relationships with the physiology of the hosts studied. The PLS model that we used transforms the primary microbiota population data into a subspace that maximally co-varies with the host responses. Using a Gaussian mixture model with bootstrap stability validation of cluster assignment, we performed unsupervised clustering of these transformed data. These studies revealed that samples are best grouped into six clusters, each of which has a distinct phenotype profile (Fig. 8a). Clusters 1, 3, and 5 were primarily associated with STAT mice. The switch from normal chow to high fat diet largely corresponds to the transition from Cluster 3 to Cluster 5. Clusters 2 and 4 were associated with Control mice receiving normal chow or HFD, respectively. As such, Cluster 4 comprises the microbiota in fecal samples primarily from 18–30-week-old mice.

To identify whether changes in host physiology are also reflected in the global rewiring of the gut microbial community structure that we observed, next we inferred microbial association networks from each of the six sample groups and analyzed their global topological network properties. Using graphlet correlation distance as a global distance measure between networks, and using isometric MDS as an analytic tool, we inferred a low-dimensional embedding of the microbial association networks (Fig. 8b). Importantly, these largely recapitulate the transitions seen in the subspace clustering described above (Fig. 8a). Networks 2 and 3 are closest to network 1. Networks 3 and 5, representing the gut microbiome community in samples from mice that received STAT are distinct from networks 2 and 4, which represent the microbial communities in samples from control mice Network 6, which is inferred from samples of older mice, is distinct from all the other networks.

Since clusters are dominated by samples that were obtained from mice under specific experimental perturbations, we classified the networks as being dominated by STAT (clusters 1, 2, and 3) or Control (2 and 4) or by normal chow (NC) (clusters 1, 2, and 3) or by HFD (4, 5, and 6). Then we calculated several graph topology statistics to assess trends as a function of sample type (Additional file 9: Figure S7). Overall, NC and STAT networks comprise more taxa, have larger network diameters, and show lower average betweenness and degree centrality. These are ecological terms indicating a node’s centrality in a network and the number of cross-ties, and low values are consistent with greater dispersion within the network. The HFD and Control networks tended to be more modular. Finally, the NC and Control networks had higher assortativity at the phylum level; this means that under normal conditions in the absence of antibiotics or an abnormal diet, particular OTUs are more likely to be directly associated with common phyla than under antibiotic and HFD exposure.

We also analyzed OTUs that could potentially serve as keystone species in the different association networks. For each network, we identified the top two taxa that serve both as hub species (having high node degree) and as bottleneck species (as characterized by the highest betweenness centrality) (Additional file 10: Figure S8). Across all networks, these potential keystone taxa are largely represented by Lactobacillus, Lachnospiraceae, and S24-7 families. For instance, in network 1, the top two taxa are *Eubacterium dolichum* and *Lactobacillus reuteri*. While these OTUs are not predicted to be directly associated with host physiological changes, both species are known to have strains that are resistant to penicillin [49, 50] and require sugar and amino acid import for survival in the host GI tract. In particular, *L. reuteri* has been shown to be a key mediator in host and microbe interactions for processing carbohydrate metabolites [51].

In addition to changes in microbial compositions, we also analyzed whether overall network robustness correlates with host health, since microbial ecological networks should have evolved to be resilient to disturbances. One example of this concept would be redundancy in network wiring that may ensure access to a vital metabolite. Thus, we hypothesized that Western-style interventions would promote network fragility by disrupting a critical threshold of keystone taxa or by changing the flux of normal metabolic exchange.

To test this hypothesis, we used natural connectivity as a general stability metric of the inferred networks after simulated network “attacks”. We found that only the network from cluster 2 – control mice receiving normal chow – was reliably robust, independent of the specific node removal strategy (Fig. 8c). Network 4, representing the microbial community after the switch to HFD, showed a decrease in network robustness, yet remained more stable than most STAT networks. Interestingly, the natural connectivity of network 4 decreased at a slower rate when bottleneck taxa were removed compared to hub taxa. This property suggests an increased redundancy of bottleneck taxa in the absence of antibiotic exposure. Importantly, microbial networks inferred in the communities in the STAT-exposed mice were found to be particularly fragile under targeted attacks, independent of the diet.

## Discussion

This study both confirms and extends our prior studies of antibiotic-induced growth promotion in murine models [11–13]. We confirm the growth promotion of STAT [12, 13] and its enhanced effect in combination with HFD compared with HFD-only controls [12]. Consistent with the enhanced adiposity were elevated plasma levels of insulin, C-peptide, leptin, and triglycerides [52]. The decreased ghrelin observed might also reflect the extreme adiposity of the mice [53]. Our observation that the perturbed microbiome is a key player in the development of NAFLD is consistent with a large body of work in both rodent models and human studies [6–10].

This work included both males and females; while the sexes shared many of the same STAT-induced phenotypes, there are several key differences in specific phenotypes related to diabetes, hyperlipidemia, and inflammatory cytokines. This model, and its manipulation, provides approaches to untangling the complex sex-specific pathophysiology observed in many prior studies [54, 55]. We do not have simple answers for the differences observed between male and female mice; however, we found differences in our prior study as well [12]. There are many prior citations in the literature showing sexual dimorphism in relation to diet and adiposity [56]. Adiposity and lean mass are primary determinants of glucose responsiveness; differences in body composition generally underlie these observations [57]. Hormonal differences between males and females may play a role in explaining these observations; and recent microbiota transfers between male and female mice affected phenotypes in a murine model of type 1 diabetes [58].

Cox et al. showed that early-life antibiotic exposure was of critical importance to the development of the obesity phenotype [12]. This study provides further supporting evidence. As previously observed [12], both body weight (Fig. 2b) and microbial community composition (Fig. 5) were already altered by the first measurement at the time of weaning (4 weeks). Furthermore, by the time of this earliest measurement, we found evidence for microbial community immaturity (Fig. 6c, d), taxa predictive of disease (Figs. 6c and 7, Additional file 8: Table S2), and altered community composition in mice that would eventually develop disease (Fig. 6c). Together, these studies further emphasize the criticality of early life microbiome perturbations in the development of later in life phenotypes, especially as enhanced by further environmental (antibiotic and/or dietary) insults. Our prior studies addressed whether a relatively brief exposure (first 4 weeks of life) was sufficient for an adiposity phenotype; we found that it was [12]. In that study, we also compared starting antibiotics slightly pre-birth and post-weaning; although the effects were in the same direction, the stronger phenotype was in the mice with the earlier exposure.

We had previously observed that female STAT mice consumed significantly more food than female control mice. In the current study (Additional file 1: Figure S1), female STAT mice consumed significantly fewer calories. Microbiome differences in the Cox et al. study (LEfSe comparison of STAT vs. control at 4 weeks) also seem inconsistent with the current study. There were a number of differences between the present studies and our previous observations, including the age at which the animals were studied in metabolic cages, and even the form of penicillin used. As such, it is hard to reach conclusions across experiments and we focus on differences within experiments in the different experimental groups.

STAT enhanced the abnormalities in insulin homeostasis observed in the mature mice, often already obese, that were receiving long-term HFD. Both male and female mice had multiple abnormal markers consistent with T2DM. Two recent, large epidemiologic studies in England and Denmark, point to prior exposure to antibiotics, even years earlier, as a risk factor for development of T2DM [59, 60]. The current studies provide a model system to more closely examine the pathogenic relationship between early life microbiome perturbation and later development of obesity and related metabolic dysfunction.

In the presence of HFD, STAT exposure causes marked hepatic abnormalities. By 32 weeks, the increased hepatic fat was visible to the unaided eye (Fig. 4a), while microscopic examination revealed marked increases in hepatic steatosis and hepatocyte ballooning (Fig. 4b, c). The fat accumulation was primarily triglycerides (Fig. 4g), with increased *Vldlr* expression in STAT (Additional file 5: Figure S4C) and diminished ABCA1 in mice with insulin resistance (Additional file 5: Figure S4D). These findings suggest that STAT-exposed mice accumulate hepatic lipids by both increased uptake (via VLDL receptor) and decreased efflux through ABCA1. One possible explanation for this phenomenon is altered gut permeability [61], allowing translocation of bacteria, their constituents, and/or their products to the hepatic parenchyma via the portal circulation; we plan to explore this hypothesis in future studies.

Since normal chow is high in plant fiber, cellulose-degrading members of the phylum Firmicutes dominate the microbial communities of control mice. As seen in this and other studies [12, 13], STAT exposure reduces Firmicutes dominance, with members of other phyla increasing in relative abundance. Obesity in humans and rodents has been associated with decreased phylogenetic diversity of the intestinal microbiota [62, 63]; however, these observations generally concerned humans and mice that already were obese. The current findings are consistent with our previous observations in STAT-exposed mice that increases in measured diversity was a predictor of the development of obesity [12]. Consistent with prior studies [12], analysis of fecal β-diversity shows that STAT-exposed communities are distinct from control from the first observation at 4 weeks, throughout life, and across all dietary interventions (Fig. 5c). LEfSe analysis showed parallel dynamic patterns in the abundance of specific taxa, in both STAT and control communities at each time point. Importantly we found that when the 4-week-old mice were grouped by outcome and not by treatment, the communities from those that would eventually develop NAFLD or insulin resistance were distinct from the communities of those who would remain healthy. One implication of this finding is that community structure in early life could be used for both prediction and for possible interventions to prevent development of metabolic diseases.

Detecting significant host-taxa associations from high-dimensional microbial compositional data, under a multi-level experimental design and with multiple, relevant clinical indications is an important challenge in microbiome research. Here, we developed a general analysis framework based on compositionally robust data transformations, data decomposition steps, and a sPLS regression that accounts for compositional biases and treatment-irrelevant variation in the data. This has led to predictions about the relationship between specific OTUs and host phenotypes, while correcting for possible colinearity within OTU and response measurements. Many of the inferred direct relationships and targeted predictions in this study are consistent with previous studies of relative abundance changes in mammalian guts.

We were also able to make novel specific predictions, e.g. that Allobaculum may increase in abundance in direct association with weight gain during aging, but still be largely protective against NAFLD, particularly in the absence of STAT. Describing gut microbiota composition in mice prone or resistant to NAFLD development, Le Roy et al. observed a negative association between Allobaculum and NAFLD, consistent with our findings [7]. The strong hepatic phenotypes were not apparent until sacrifice, so we did not have the opportunity to explore them pre-mortem. However, we found high NAFLD activity scores (Fig. 4c) and substantial inflammation (Fig. 4d), and were able to characterize the nature of the lipid accumulation (Fig. 4f–h). Future studies will focus on specific steps leading to these extreme phenotypes.

We had previously observed significant differences in bone mineral density due to STAT exposures [12, 13]. To address this point further, we sought to determine whether microbiota composition would predict scale weight at the next measured time point. Although using the PLS model, there are differences between Weight and Weight + 1, we did not quantify these relatively minor effects. On the other hand, including Weight + 1 led to a stable clustering solution; therefore, we kept this response variable for consistency while developing the pipeline.

The proposed analysis techniques also have the power to correct for transitive correlations, e.g. by distinguishing between direct and indirect associations between specific SFB OTUs and NAFLD or other body composition measurements. Additionally, we have demonstrated that learning OTU-OTU associations in different ecological contexts can lead to predictions about how entire ecosystems are structured and to identification of keystone species. While these species may be distinct from those that demonstrably co-vary with host phenotypes, they could be critical control points through which ecological interventions propagate. For instance, we have found that non-intervention corresponds to overall network stability, even in an inbred mouse strain, but targeted removal of critical nodes in the presence of low doses of antibiotics could lead to ecosystem collapse. These putative keystone taxa: *E. dolichum* and, in particular *L. reuteri*, have been shown to have probiotic effects and many microbe interactions. We confirm this finding with network analysis and additionally postulate that these taxa could be the last line of defense in the presence of a significant intervention (STAT). However, before targeted experiments can be done, we must identify species and strain level identities for these taxa, as well as construct dynamic models, which requires more densely sampled time series. The first point guarantees specificity of a transfer or targeted intervention, while the second point would allow us to generate hypotheses about the direction and magnitude of the impact.

## Conclusions

In conclusion, extension of the STAT studies provides new models relevant to the pathogenesis of obesity, T2D, and NAFLD. The consistency of the observations, both internally and in relation to prior studies [12, 13], indicate the tractability of the model for future investigations. Use of perturbations, such as dietary and antibiotic exposures, and developing new computational tools provides new approaches for assessing the complexity inherent in studies of the relationship between the gut microbiota and metabolic phenotypes and disease.

### Ethics approval

All animal experiments were performed according to IACUC-approved protocols.

### Availability of data and materials

The 16S sequence data have been uploaded to Qiita (https://qiita.ucsd.edu/) with Study ID: 10469 as the identifier. These data are also available on EBI (https://www.ebi.ac.uk/metagenomics/) with ERP014859 as the identifier.

## Additional files

Additional file 1: Figure S1. STAT did not change energy balance, or bone morphometrics. STAT (3 male, 3 female) and control (3 male, 3 female) mice were housed individually in metabolic cages for 5 days. **A** Gross caloric intake. Food consumed was measured daily by weight. Calories per gram from the chow package insert were used to calculate calories consumed. Each point represents a single 24 h of data. **B** Gross fecal calories. All fecal pellets were harvested for each mouse each day, and homogenized to create a daily fecal pellet for each mouse. Total dry fecal volume per day combined with bomb calorimeter values for the daily pellets were used to determine total calories out. **C** Net daily calories, calculated as (calories in (from A) – calories out (from B)). **D** Proportion of daily caloric intake retained (Net daily calories (from C) divided by gross caloric intake (from A)). Data points are the mean of duplicate determinations (Kruskal–Wallis test; **p* <0.05). DEXA bone measurements for all four groups: **E** mineral density, **F** mineral content, and **G** area. Arrow indicates start of the HFD. *p* values calculated from individual mouse data (Mann–Whitney *U* test; **p* <0.05; ***p* <0.01; ****p* <0.001). Gray dots signify outliers greater than 2 standard deviations from the mean. (PDF 1337 kb) Additional file 2: Figure S2. Quality scores across all sequenced bases. Amplicon pools were sequenced on the 2 × 150 bp Illumina MiSeq platform and total reads were across both paired ends. **A** For 10, 725,612 total reads, 3,688,882 were joined using the EA-utils toolbox. To ensure higher fidelity of paired-end read-joining, bases with PHRED threshold <18 were trimmed from the ends of each sequence read (number of “Trimmed Seqs” for each paired end read). **B**, **C** With summarization produced by FastQC, the average and range of base-pair quality was high (PHRED scores >30) across the length of the sequenced reads. (PDF 89 kb) Additional file 3: Table S1. Adonis tests of repeated measures. (DOCX 20 kb) Additional file 4: Figure S3. A We used the mclust package to find the optimal model and number of mixture parameters (number of data classes) using a 14 variants of a Gaussian mixture model (GMM), over 3-9 possible data classes. The VVE model (ellipsoidal, equal orientation) under 6 latent components was selected, under a Bayesian Information Criterion (starred). The EVE model (ellipsoidal, equal volume and orientation) was also competitive, but slightly suboptimal. For a full list of possible models, see the help page for ’mclustModelNames’ in R. B To visualize the clustering solution, we back-transformed the data (sPLS scores) using the within-cluster precision matrix and scaling factor, estimated by the GMM independently for each cluster. The first three model components are shown, together with cluster identity and Control/STAT status. (PDF 65 kb) Additional file 5: Figure S4. Inflammatory and metabolic profiling in control and STAT mice. Serum was collected at 32 weeks for analysis by MILLIPLEX® MAP Magnetic Bead Panel. Circulating inflammatory hormones **A** IL-6 and **B** TNF-α were measured. Mice were sacrificed at 32 weeks of age and hepatic gene expression was assessed using RT-qPCR. **C** Genes involved in free fatty acid (FFA) transport, triglyceride (TG) uptake, fatty acid catabolism, TG synthesis, lipid droplet formation, transcription factors, and FFA oxidation were measured. **D** *Abca1* expression from mice that had normal vs. high HOMA-IR scores. **E** *Cd36* expression from mice that had normal vs. high HOMA-IR scores. **F** *Cd36* expression from mice that had normal vs. positive NAFLD diagnosis. *p* values calculated from individual mouse data (Mann–Whitney *U* test; **p* <0.05). (PDF 64 kb) Additional file 6: Figure S5. STAT alters microbial communities. Unifrac distance at weeks 4, 11, 16, and 3. **A** Control (C) vs. STAT (S); “*I*” represents intergroup measures. **B** Healthy (I) vs. disease (D) outcome; “*I*” represents intergroup measures. **C** Control (C) vs. STAT (S) with non-responder females; “*CN*” represents the intergroup measure between control and non-responders; “*NS*” represents the intergroup distance between non-responders and STAT. *p* values calculated by Kruskal–Wallis and AUC analysis (**p* <0.05; ***p* <0.01; ****p* <0.001). (PDF 58 kb) Additional file 7: Figure S6. Additional OTU-host phenotype associations. **A** Isometric Multidimensional scaling (MDS) of Euclidean distance between CLR transformed OTU compositions. The first two MDS components are shown, with Controls vs. STAT and NC vs. HFD (point color, shape) leads to an effective unsupervised grouping. **B** Additional biplots for within-subject response-selected OTUs are shown. For each phenotype of interest (DMI, Fat, Lean, Weight + 1), the relevant two component subspace from the sPLS model (out of six possible latent components) are shown. Taxa are filtered for statistical significance (α = 10^–2^) and key taxa are highlighted for biological significance. “Response Level” indicates the centered and scaled within-subject variances of the relevant measurement. (PDF 125 kb) Additional file 8: Table S2. Significant Taxa-host associations as determined from sparse PLS regression. *p* values are based on bootstrap evaluation of the PLS permutation test compared to a permutation-based null model on the variable support selected by sparse PLS. (PDF 43 kb) Additional file 9: Figure S7. Since clusters are dominated by samples under specific experimental perturbations, we classified networks as being dominated by STAT (clusters 1, 2 and 3) or Control (2 and 4) or by NC (clusters 1, 2 and 3) or HFD (4, 5, and 6). We computed a number of graph topology statistics to assess trends as a function of sample type. (PDF 70 kb) Additional file 10: Figure S8. Betweenness centrality vs. Degree. For each node, we computed the degree and betweenness centrality. The top two OTUs by betweenness are consistent with degree, and are highlighted in gray. For each network the lineages of these are: 1. f__Erysipelotrichaceae;g__[Eubacterium];s__dolichum and f__Lactobacillaceae;g__Lactobacillus;s__reuteri; 2. o__Clostridiales and o__Clostridiales;f__Ruminococcaceae;g__Oscillospira; 3. o__Bacteroidales;f__S24-7 and o__Clostridiales;f__Lachnospiraceae; 4. o__Bacteroidales;f__S24-7 and o__Clostridiales;f__Dehalobacteriaceae;g__Dehalobacterium; 5. o__Clostridiales;f__Lachnospiraceae;g__Roseburia and o__Clostridiales; 6. o__Bacteroidales;f__S24-7 and f__Lactobacillaceae;g__Lactobacillus. (PDF 301 kb)

## Abbreviations

clr: Centered log-ratio
DEXA: Dual energy X-ray absorptiometry
FnR: Female non-responders
HFD: High-fat diet
HOMA-IR: Homeostatic model assessment of insulin resistance
IPGTT: Intraperitoneal (IP) glucose tolerance tests
IPITT: Intraperitoneal insulin tolerance tests
ISOMDS: Isometric multidimensional scaling
LEfSe: Linear discriminant analysis effect size
MDS: Multidimensional scaling
NAFLD: Non-alcoholic fatty liver disease
pam: Partitioning around mediods
PCoA: Principal coordinates analysis
SPIEC-EASI: Sparse Inverse Covariance estimation for Ecological ASsociation Inference
sPLS: L1-penalized partial least squares regression
StARS: Stability approach to regularization selection
STAT: Sub-therapeutic antibiotic treatment
T2DM: Type 2 diabetes
