# Supplementary figures and images for: Antibiotic perturbation of the murine gut microbiome enhances the adiposity, insulin resistance, and liver disease associated with high-fat diet
Source: Genome Med. 2016 Apr 27;8:48. doi: 10.1186/s13073-016-0297-9 (PMC4847194; doi:10.1186/s13073-016-0297-9)

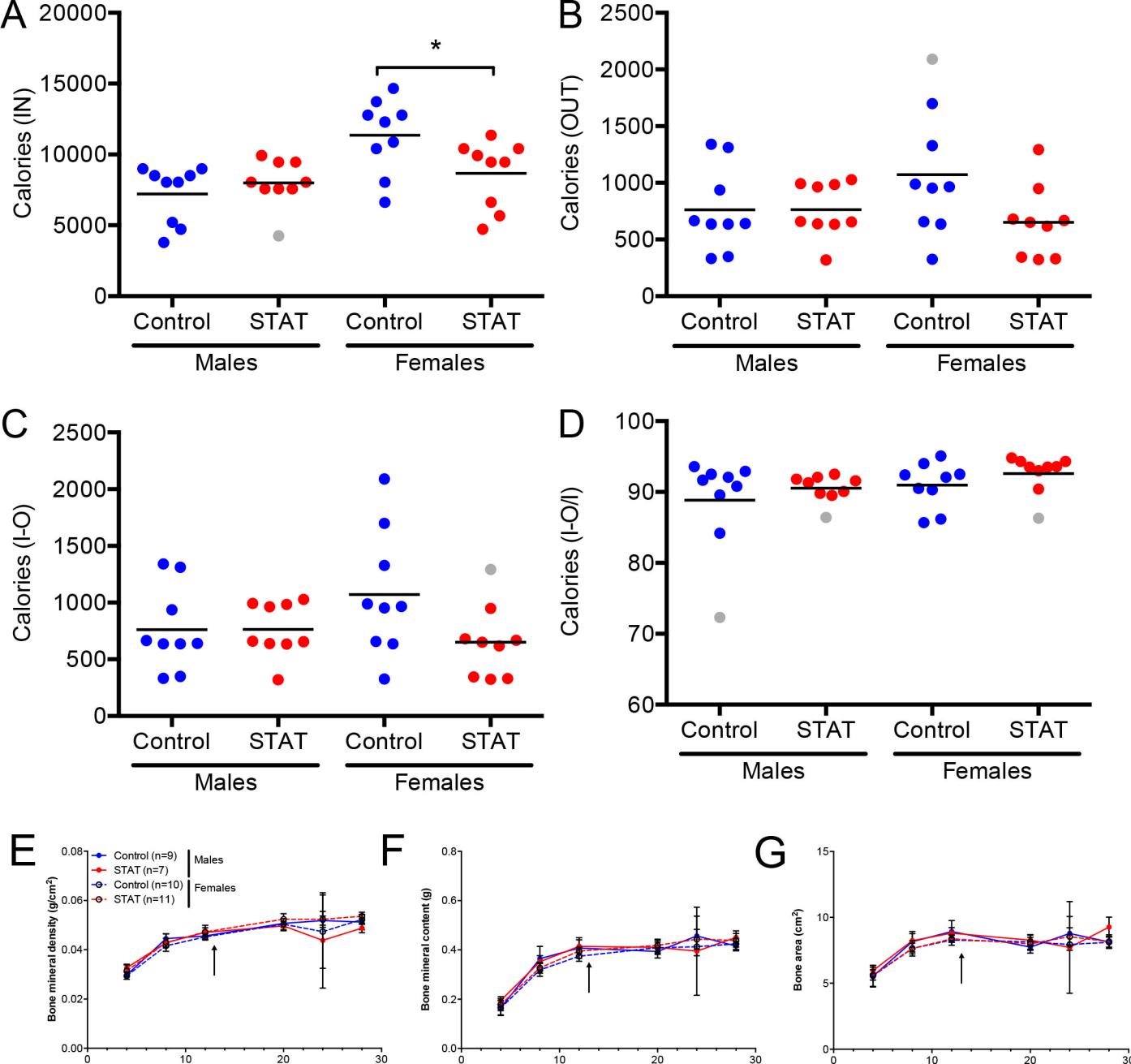

Supplement: Additional file 1: Figure S1. — STAT did not change energy balance, or bone morphometrics. STAT (3 male, 3 female) and control (3 male, 3 female) mice were housed individually in metabolic cages for 5 days. A Gross caloric intake. Food consumed was measured daily by weight. Calories per gram from the chow package insert were used to calculate calories consumed. Each point represents a single 24 h of data. B Gross fecal calories. All fecal pellets were harvested for each mouse each day, and homogenized to create a daily fecal pellet for each mouse. Total dry fecal volume per day combined with bomb calorimeter values for the daily pellets were used to determine total calories out. C Net daily calories, calculated as (calories in (from A) – calories out (from B)). D Proportion of daily caloric intake retained (Net daily calories (from C) divided by gross caloric intake (from A)). Data points are the mean of duplicate determinations (Kruskal–Wallis test; *p <0.05). DEXA bone measurements for all four groups: E mineral density, F mineral content, and G area. Arrow indicates start of the HFD. p values calculated from individual mouse data (Mann–Whitney U test; *p <0.05; **p <0.01; ***p <0.001). Gray dots signify outliers greater than 2 standard deviations from the mean. (PDF 1337 kb) [file 13073_2016_297_MOESM1_ESM.pdf]

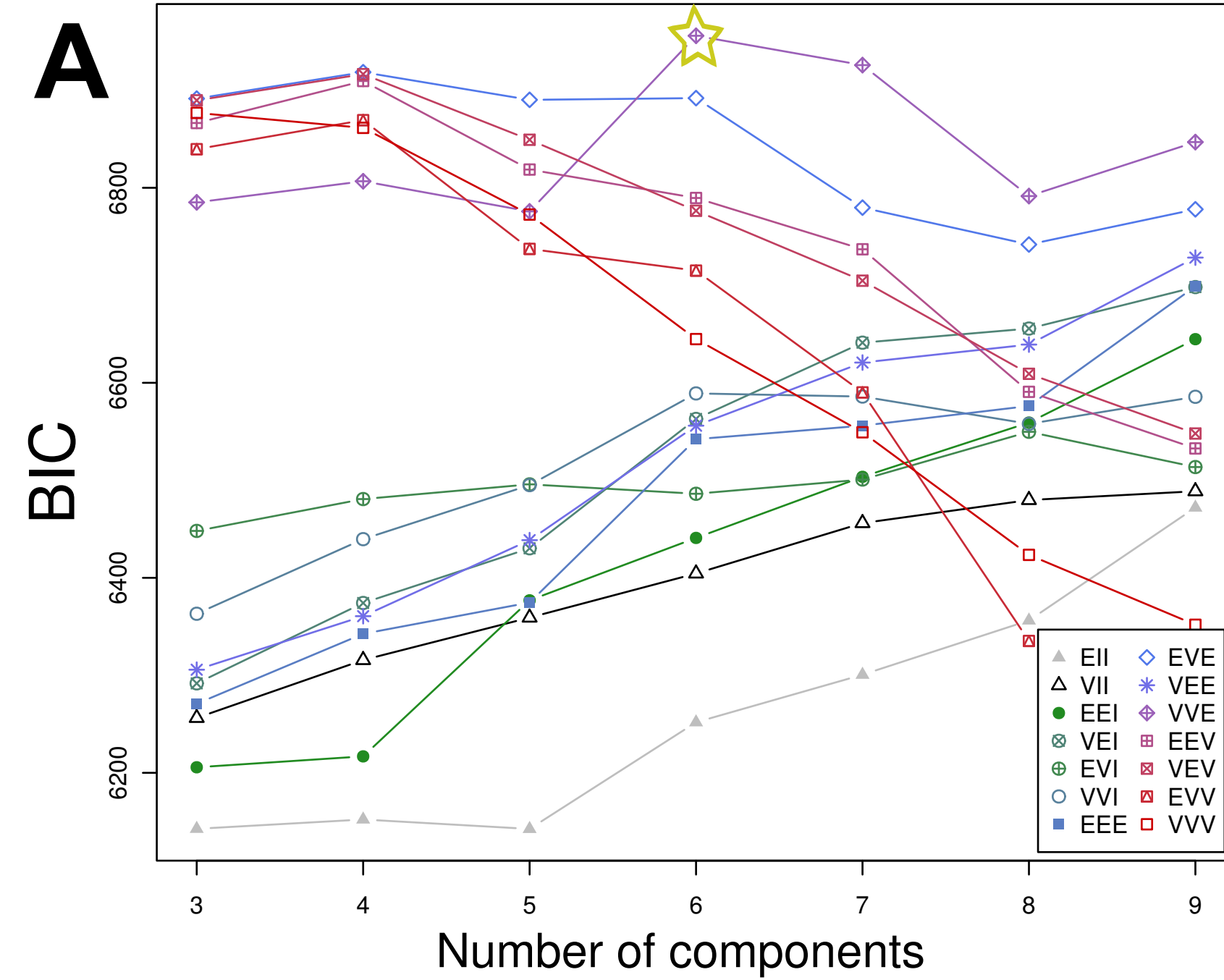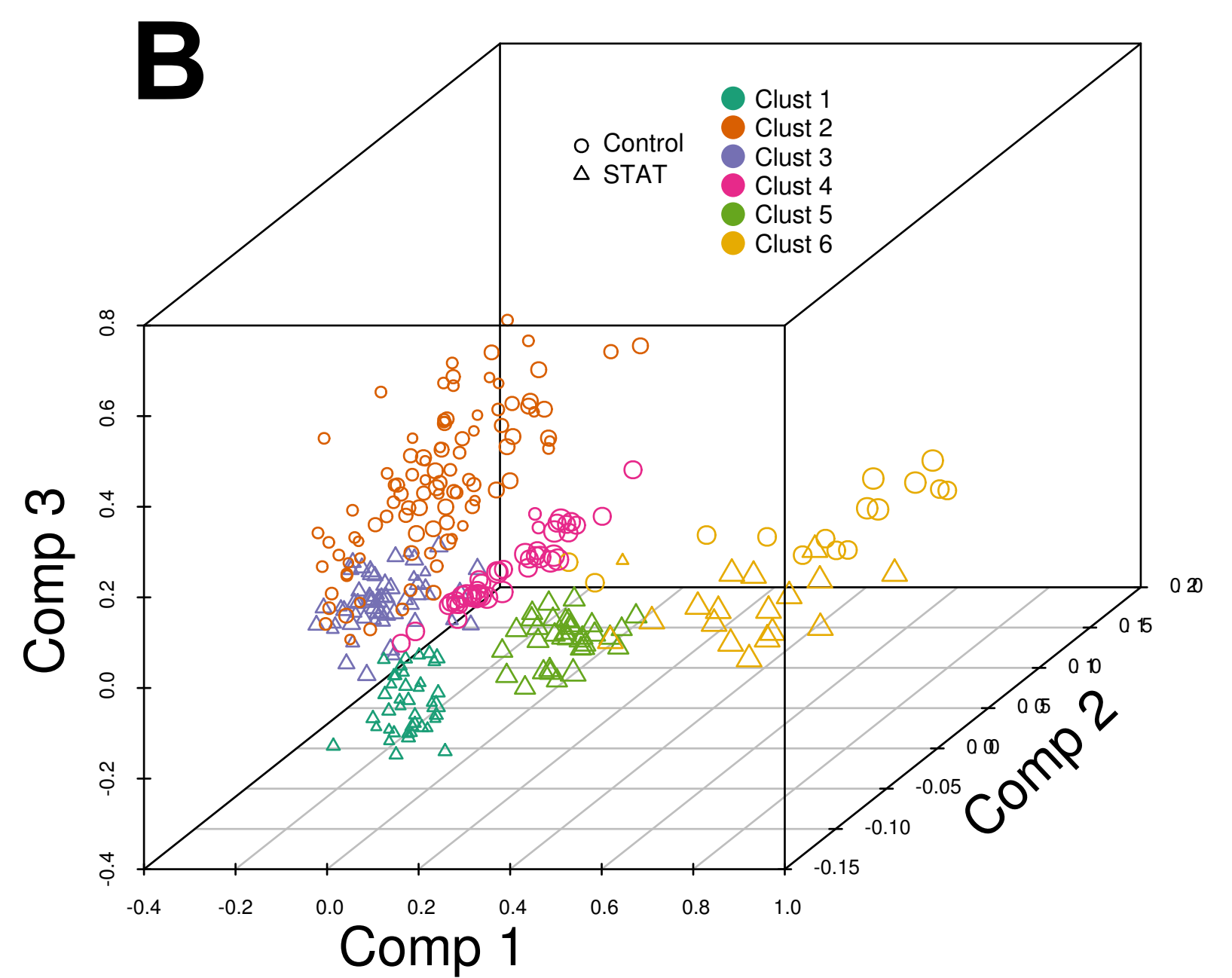

Supplement: Additional file 4: Figure S3. — A We used the mclust package to find the optimal model and number of mixture parameters (number of data classes) using a 14 variants of a Gaussian mixture model (GMM), over 3-9 possible data classes. The VVE model (ellipsoidal, equal orientation) under 6 latent components was selected, under a Bayesian Information Criterion (starred). The EVE model (ellipsoidal, equal volume and orientation) was also competitive, but slightly suboptimal. For a full list of possible models, see the help page for ’mclustModelNames’ in R. B To visualize the clustering solution, we back-transformed the data (sPLS scores) using the within-cluster precision matrix and scaling factor, estimated by the GMM independently for each cluster. The first three model components are shown, together with cluster identity and Control/STAT status. (PDF 65 kb) [file 13073_2016_297_MOESM4_ESM.pdf]

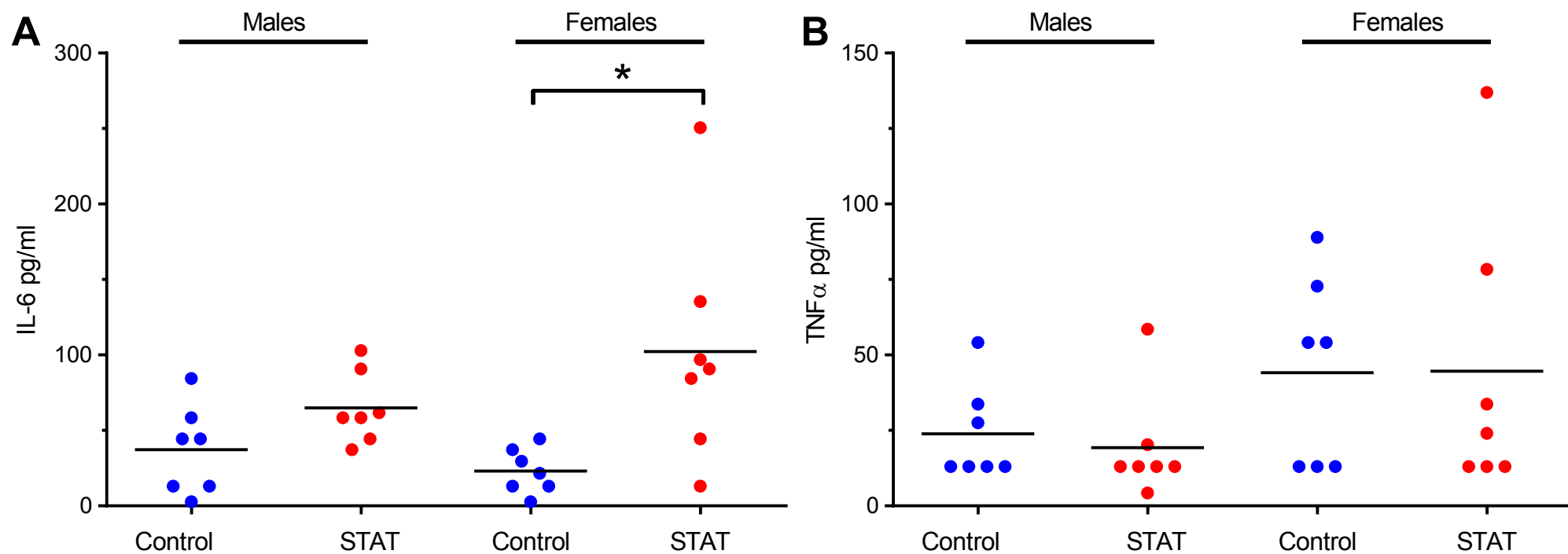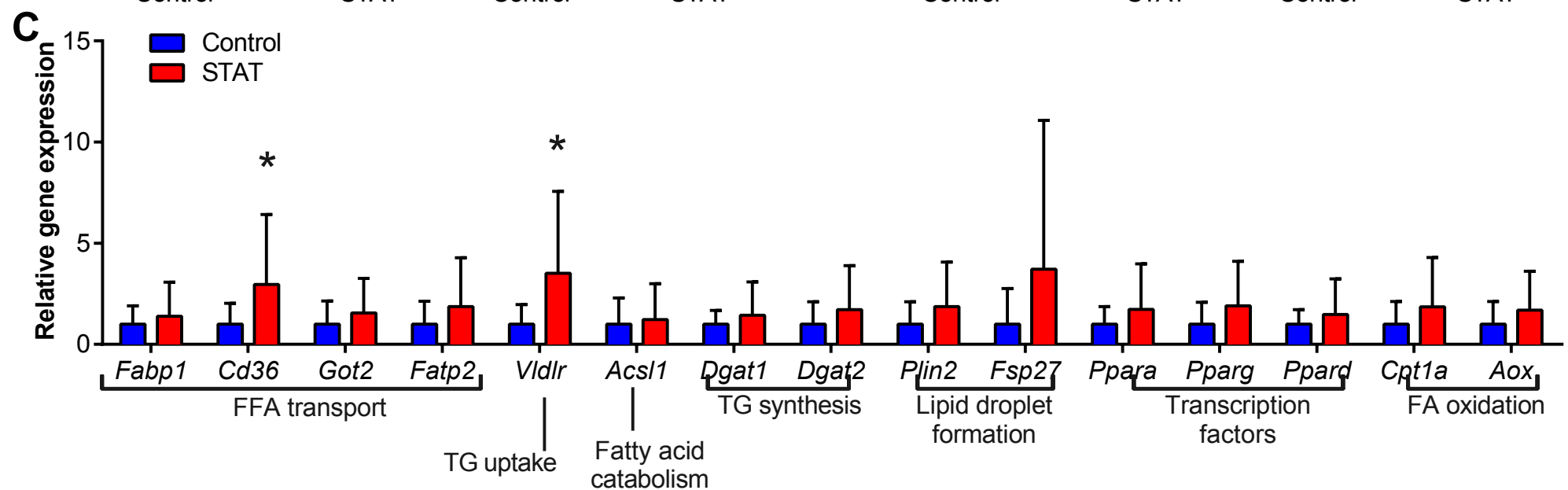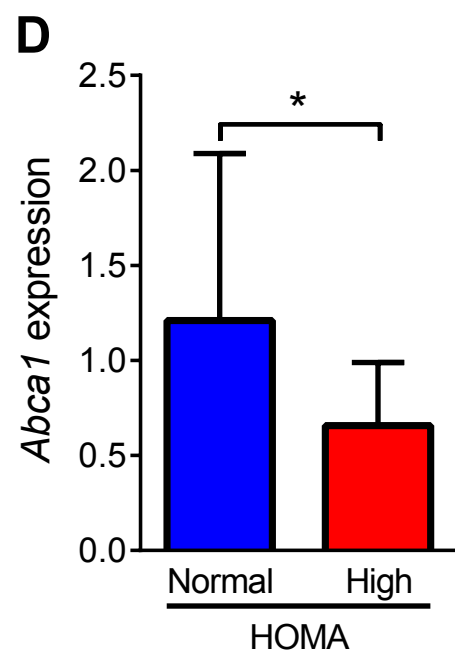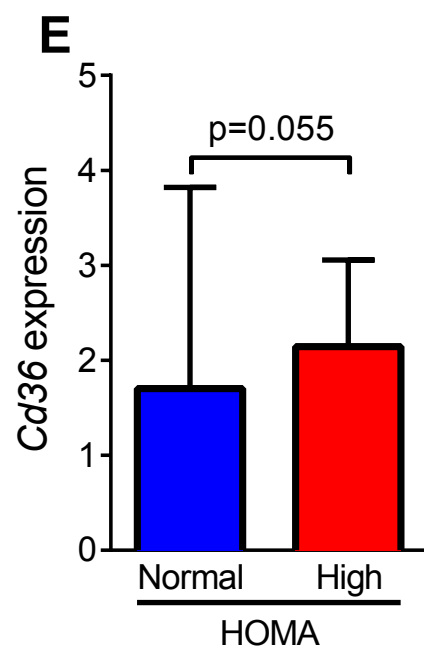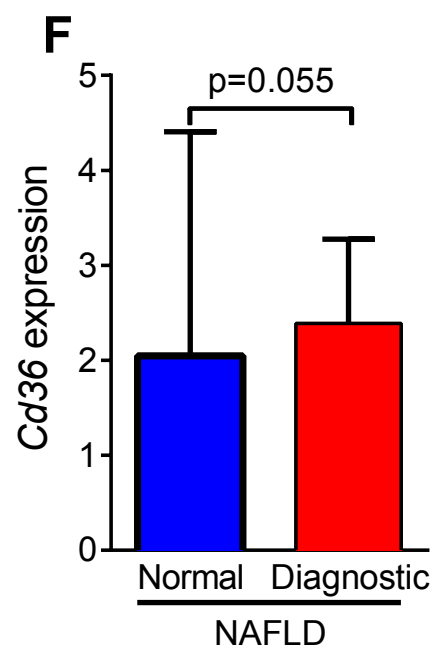

Supplement: Additional file 5: Figure S4. — Inflammatory and metabolic profiling in control and STAT mice. Serum was collected at 32 weeks for analysis by MILLIPLEX® MAP Magnetic Bead Panel. Circulating inflammatory hormones A IL-6 and B TNF-α were measured. Mice were sacrificed at 32 weeks of age and hepatic gene expression was assessed using RT-qPCR. C Genes involved in free fatty acid (FFA) transport, triglyceride (TG) uptake, fatty acid catabolism, TG synthesis, lipid droplet formation, transcription factors, and FFA oxidation were measured. D Abca1 expression from mice that had normal vs. high HOMA-IR scores. E Cd36 expression from mice that had normal vs. high HOMA-IR scores. F Cd36 expression from mice that had normal vs. positive NAFLD diagnosis. p values calculated from individual mouse data (Mann–Whitney U test; *p <0.05). (PDF 64 kb) [file 13073_2016_297_MOESM5_ESM.pdf]

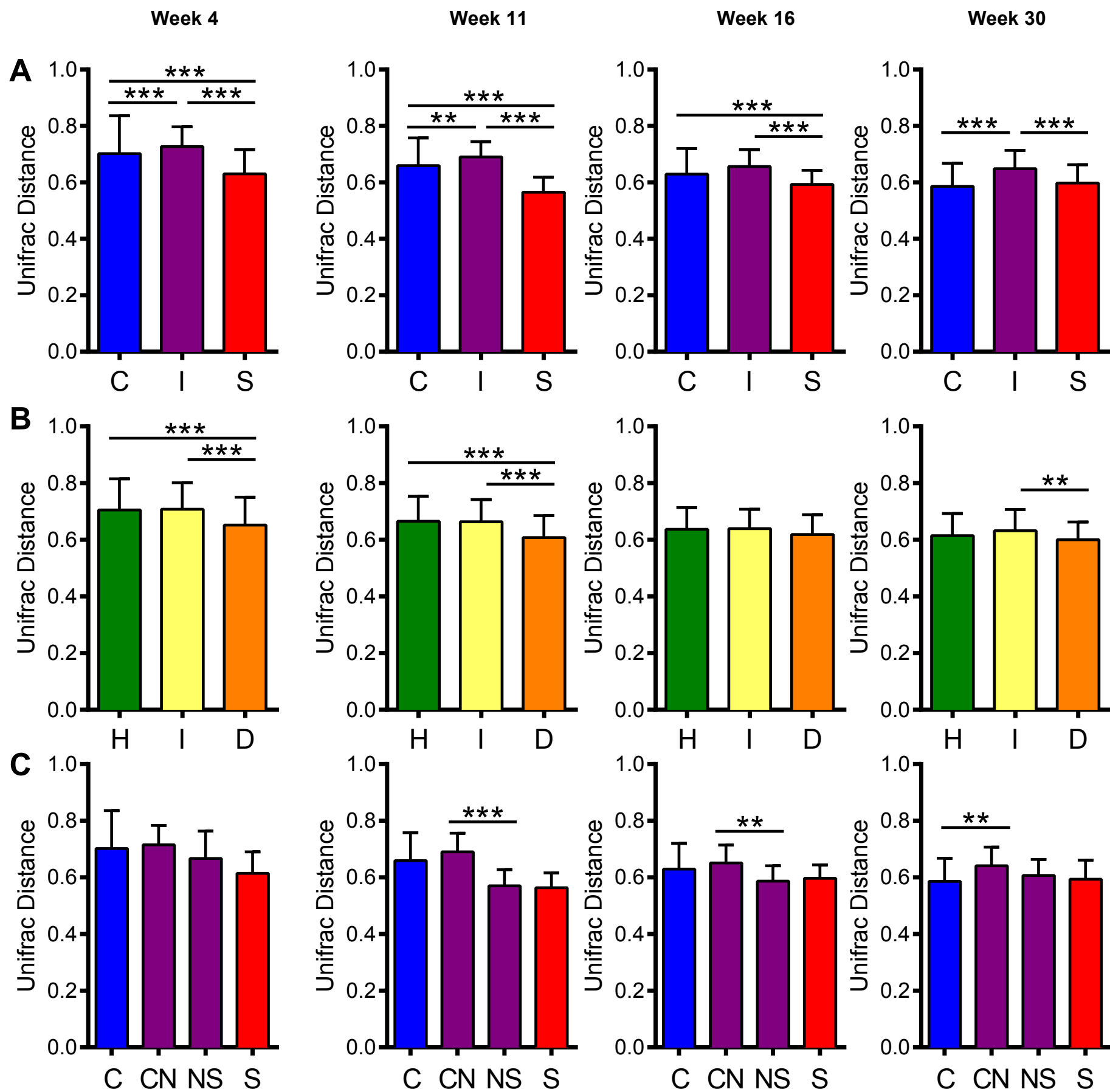

Supplement: Additional file 6: Figure S5. — STAT alters microbial communities. Unifrac distance at weeks 4, 11, 16, and 3. A Control (C) vs. STAT (S); “I” represents intergroup measures. B Healthy (I) vs. disease (D) outcome; “I” represents intergroup measures. C Control (C) vs. STAT (S) with non-responder females; “CN” represents the intergroup measure between control and non-responders; “NS” represents the intergroup distance between non-responders and STAT. p values calculated by Kruskal–Wallis and AUC analysis (*p <0.05; **p <0.01; ***p <0.001). (PDF 58 kb) [file 13073_2016_297_MOESM6_ESM.pdf]

**A**

ISOMDS of CLR, stress 26.3

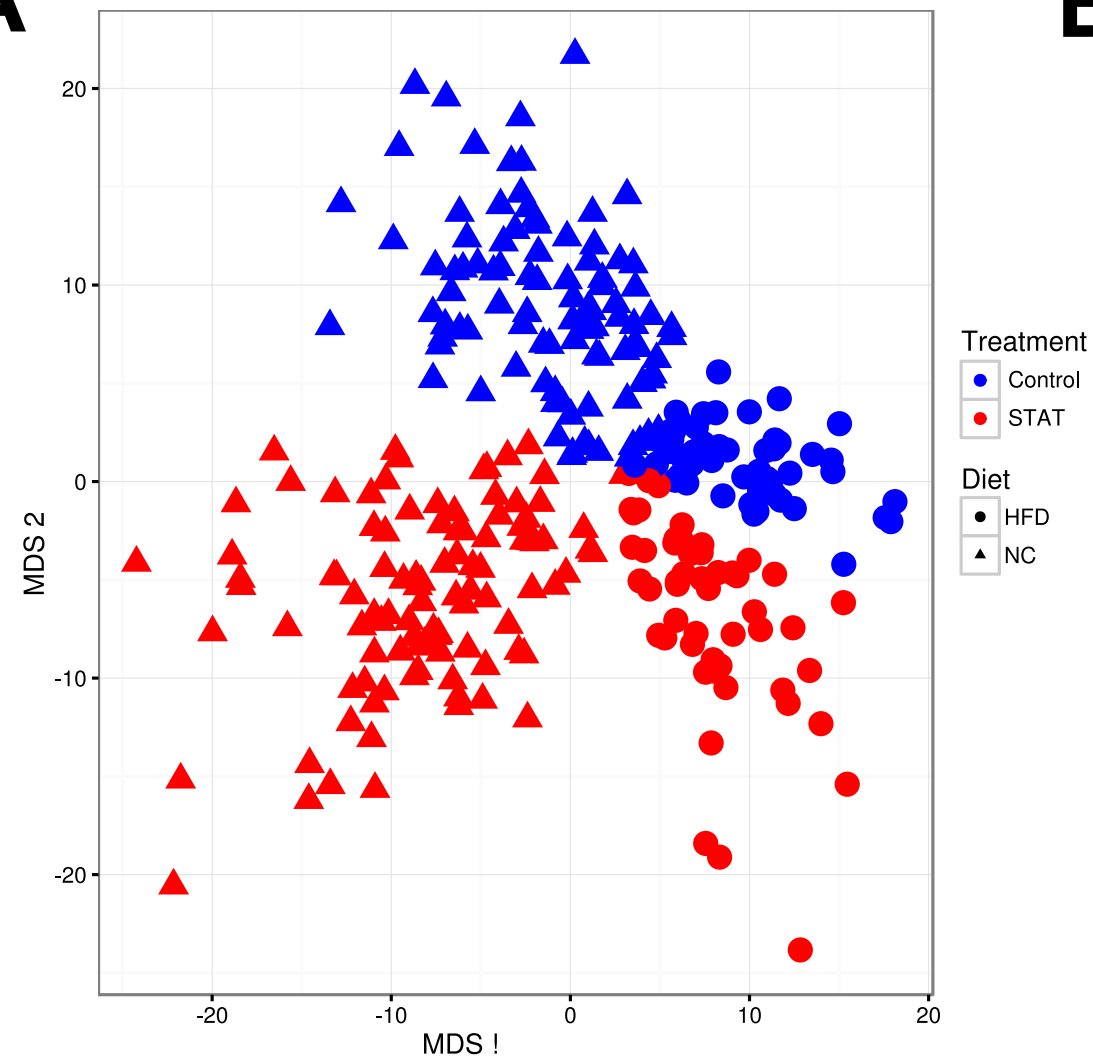

OTU at Phylum level

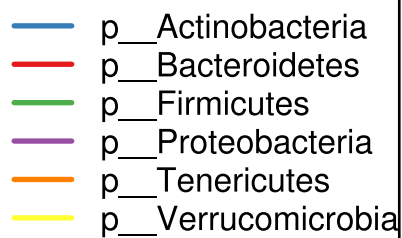**B**

DMI

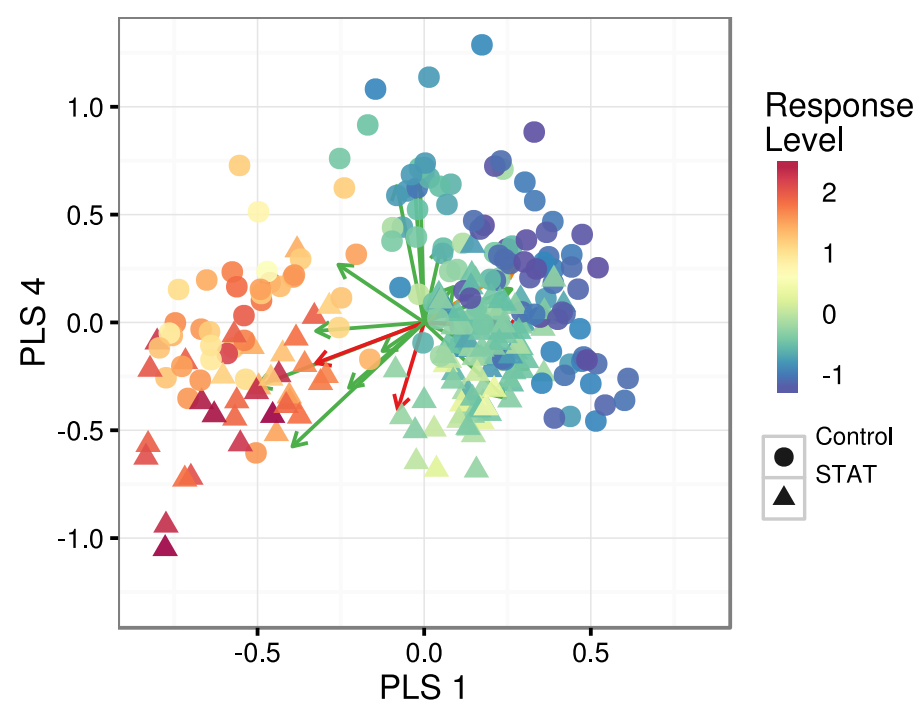

Fat

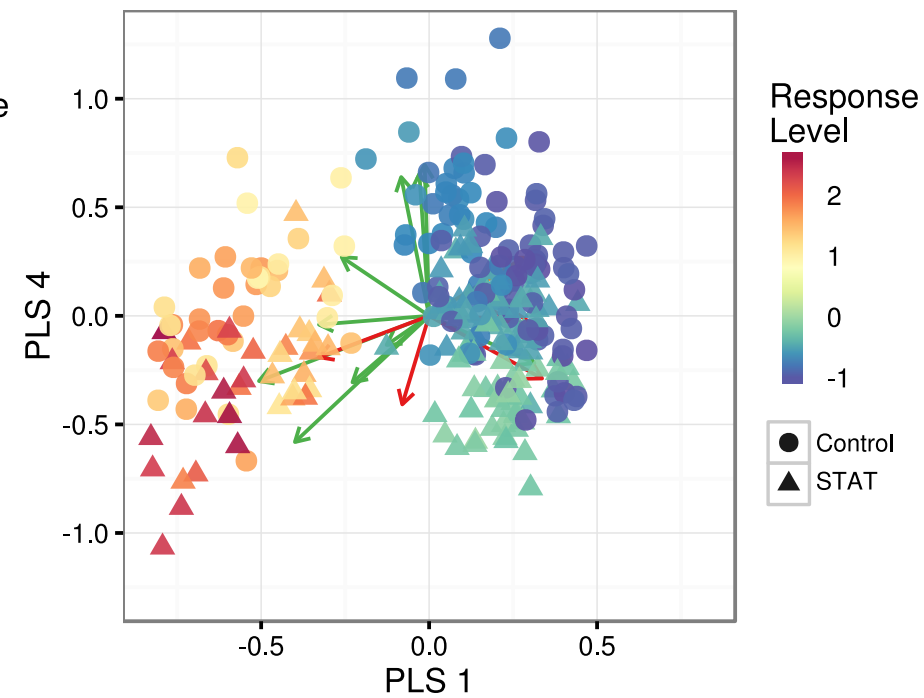

Lean

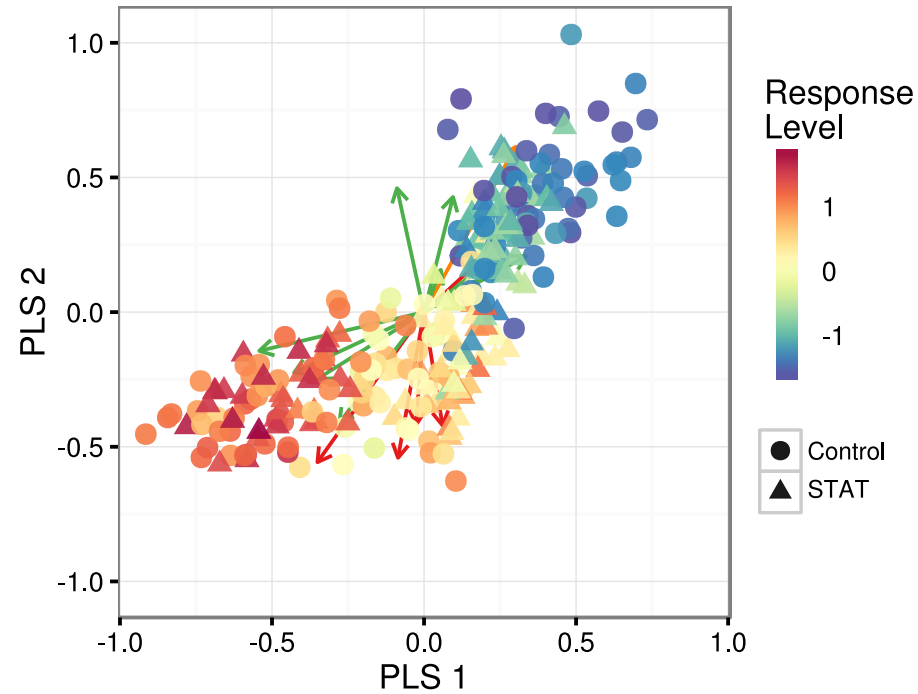

Weight+1

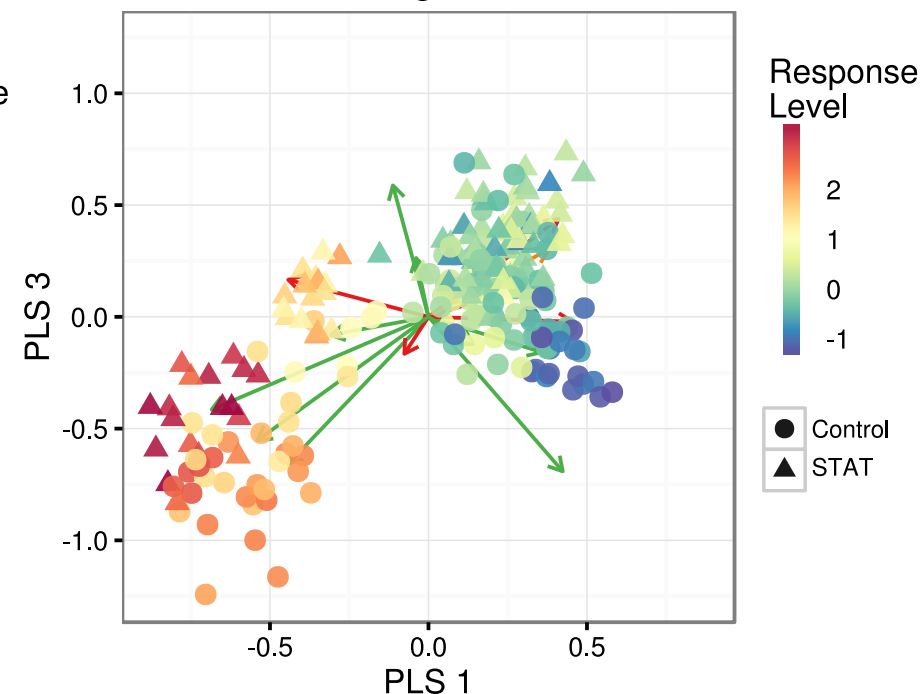

Supplement: Additional file 7: Figure S6. — Additional OTU-host phenotype associations. A Isometric Multidimensional scaling (MDS) of Euclidean distance between CLR transformed OTU compositions. The first two MDS components are shown, with Controls vs. STAT and NC vs. HFD (point color, shape) leads to an effective unsupervised grouping. B Additional biplots for within-subject response-selected OTUs are shown. For each phenotype of interest (DMI, Fat, Lean, Weight + 1), the relevant two component subspace from the sPLS model (out of six possible latent components) are shown. Taxa are filtered for statistical significance (α = 10–2) and key taxa are highlighted for biological significance. “Response Level” indicates the centered and scaled within-subject variances of the relevant measurement. (PDF 125 kb) [file 13073_2016_297_MOESM7_ESM.pdf]

**A**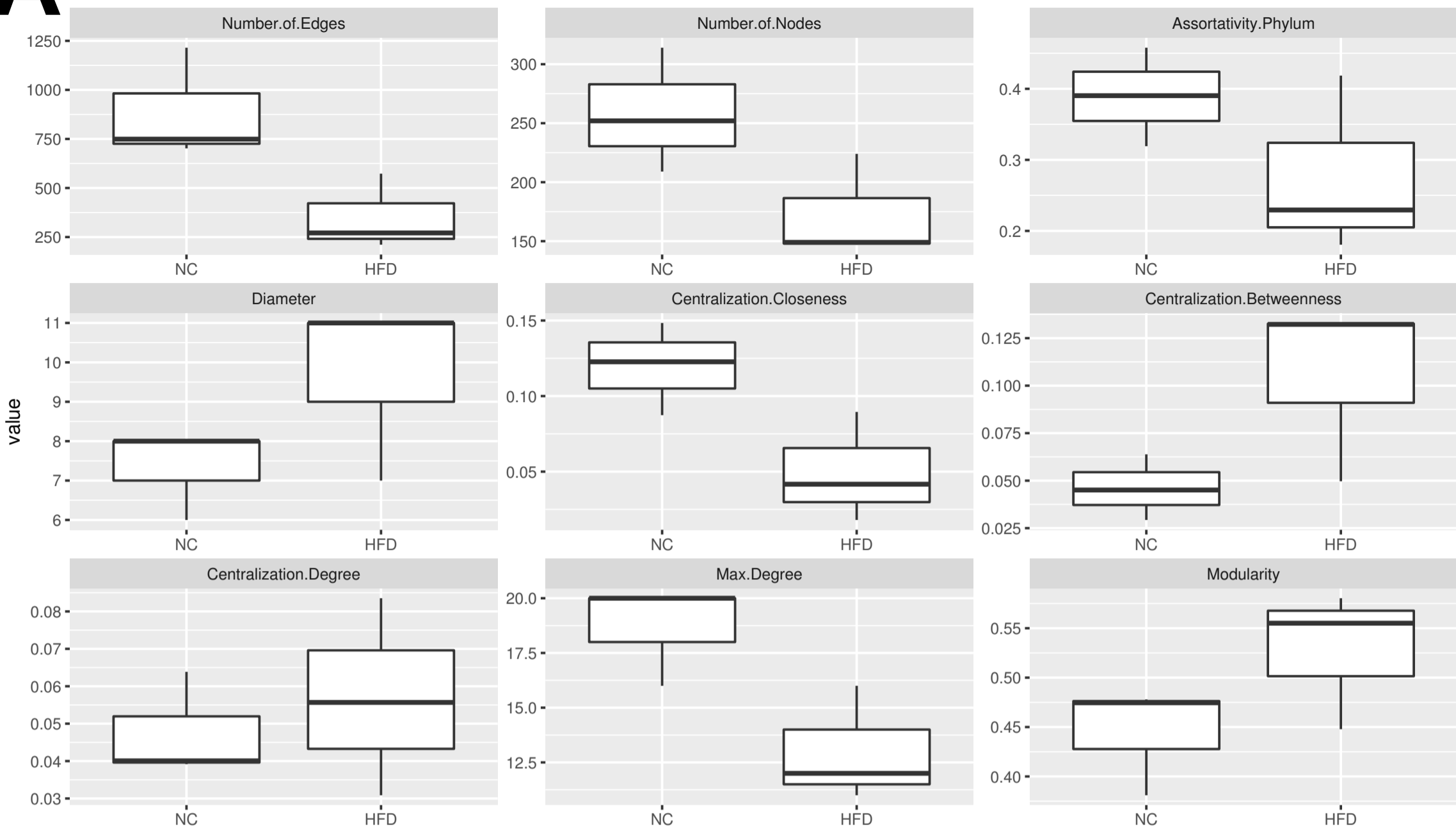**B**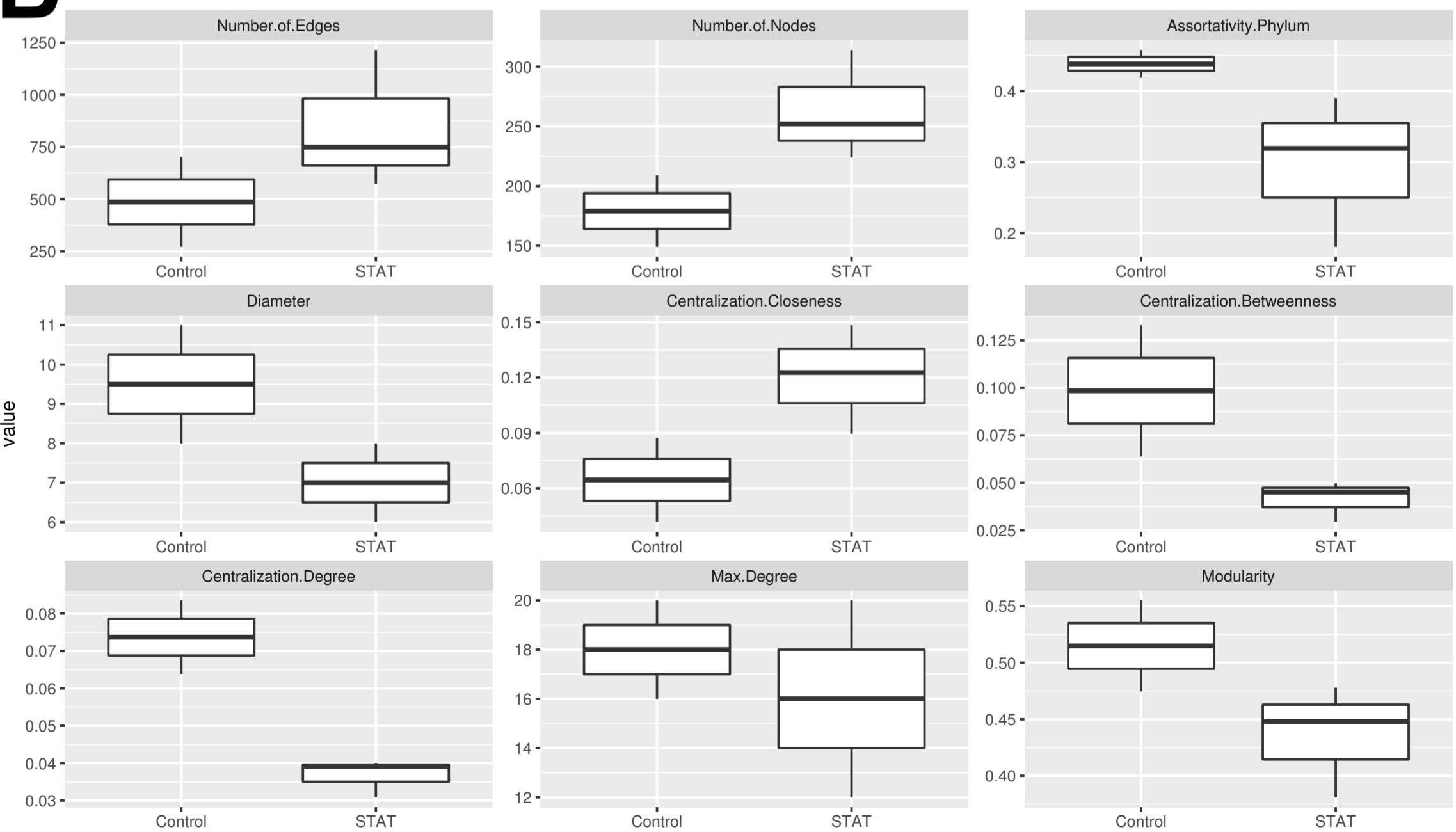

Supplement: Additional file 9: Figure S7. — Since clusters are dominated by samples under specific experimental perturbations, we classified networks as being dominated by STAT (clusters 1, 2 and 3) or Control (2 and 4) or by NC (clusters 1, 2 and 3) or HFD (4, 5, and 6). We computed a number of graph topology statistics to assess trends as a function of sample type. (PDF 70 kb) [file 13073_2016_297_MOESM9_ESM.pdf]

Network 1

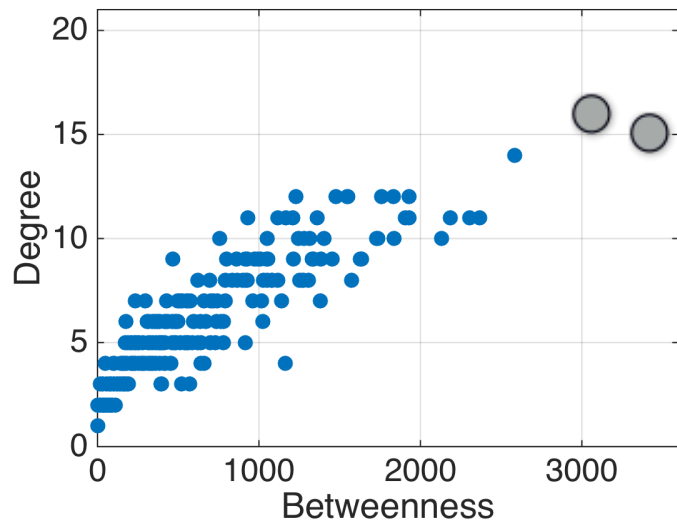

Network 2

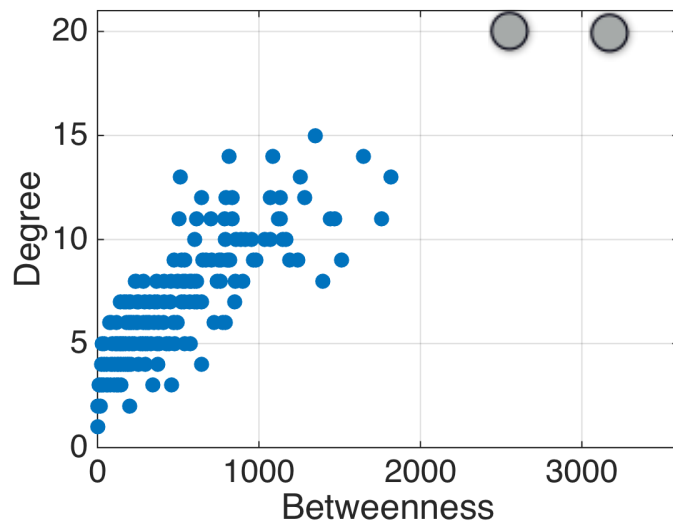

Network 3

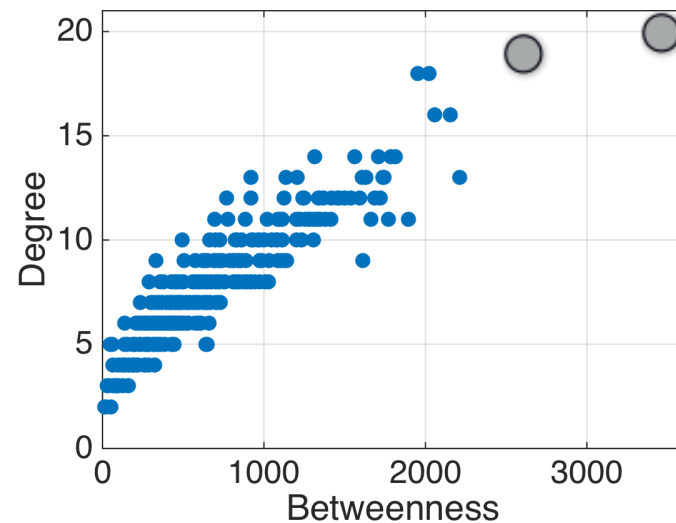

Network 4

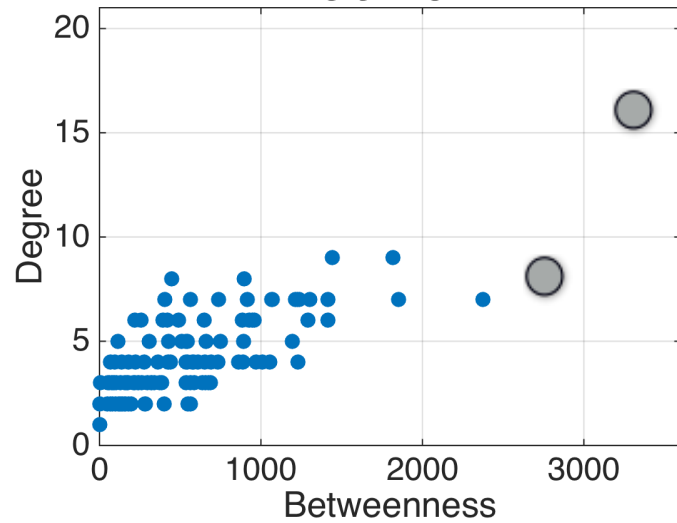

Network 5

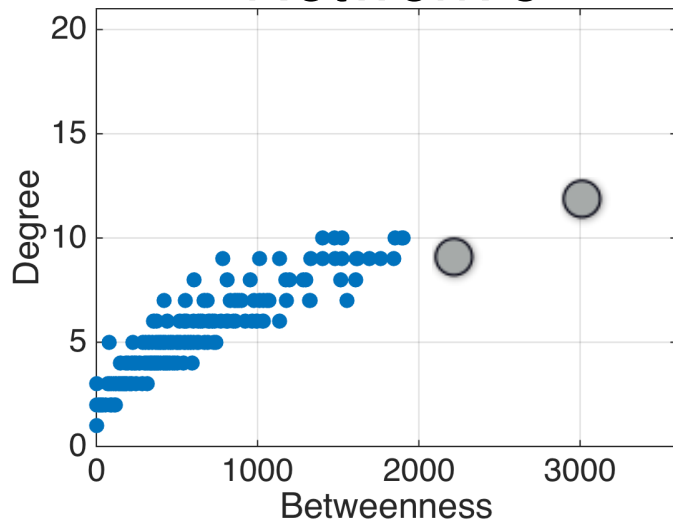

Network 6

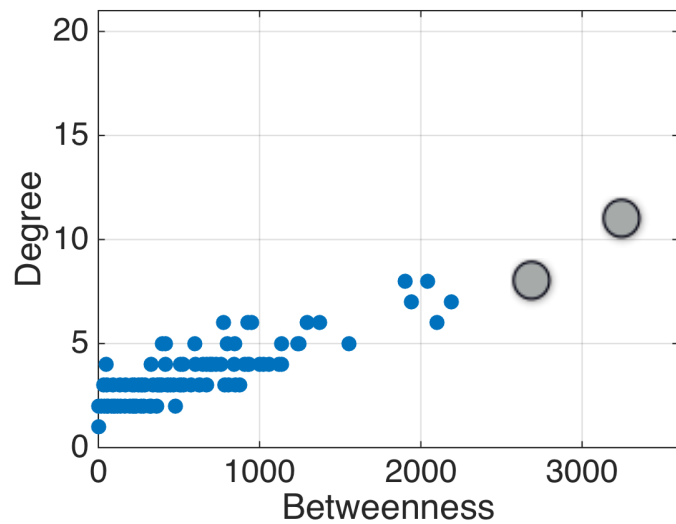

Supplement: Additional file 10: Figure S8. — Betweenness centrality vs. Degree. For each node, we computed the degree and betweenness centrality. The top two OTUs by betweenness are consistent with degree, and are highlighted in gray. For each network the lineages of these are: 1. f__Erysipelotrichaceae;g__[Eubacterium];s__dolichum and f__Lactobacillaceae;g__Lactobacillus;s__reuteri; 2. o__Clostridiales and o__Clostridiales;f__Ruminococcaceae;g__Oscillospira; 3. o__Bacteroidales;f__S24-7 and o__Clostridiales;f__Lachnospiraceae; 4. o__Bacteroidales;f__S24-7 and o__Clostridiales;f__Dehalobacteriaceae;g__Dehalobacterium; 5. o__Clostridiales;f__Lachnospiraceae;g__Roseburia and o__Clostridiales; 6. o__Bacteroidales;f__S24-7 and f__Lactobacillaceae;g__Lactobacillus. (PDF 301 kb) [file 13073_2016_297_MOESM10_ESM.pdf]
