# Supplementary material for: Antibiotic perturbation of the murine gut microbiome enhances the adiposity, insulin resistance, and liver disease associated with high-fat diet
Source: Genome Med. 2016 Apr 27;8:48. doi: 10.1186/s13073-016-0297-9 (PMC4847194; doi:10.1186/s13073-016-0297-9)
Supplement: Additional file 2: Figure S2. — Quality scores across all sequenced bases. Amplicon pools were sequenced on the 2 × 150 bp Illumina MiSeq platform and total reads were across both paired ends. A For 10, 725,612 total reads, 3,688,882 were joined using the EA-utils toolbox. To ensure higher fidelity of paired-end read-joining, bases with PHRED threshold <18 were trimmed from the ends of each sequence read (number of “Trimmed Seqs” for each paired end read). B, C With summarization produced by FastQC, the average and range of base-pair quality was high (PHRED scores >30) across the length of the sequenced reads. (PDF 89 kb) [file 13073_2016_297_MOESM2_ESM.pdf]

C

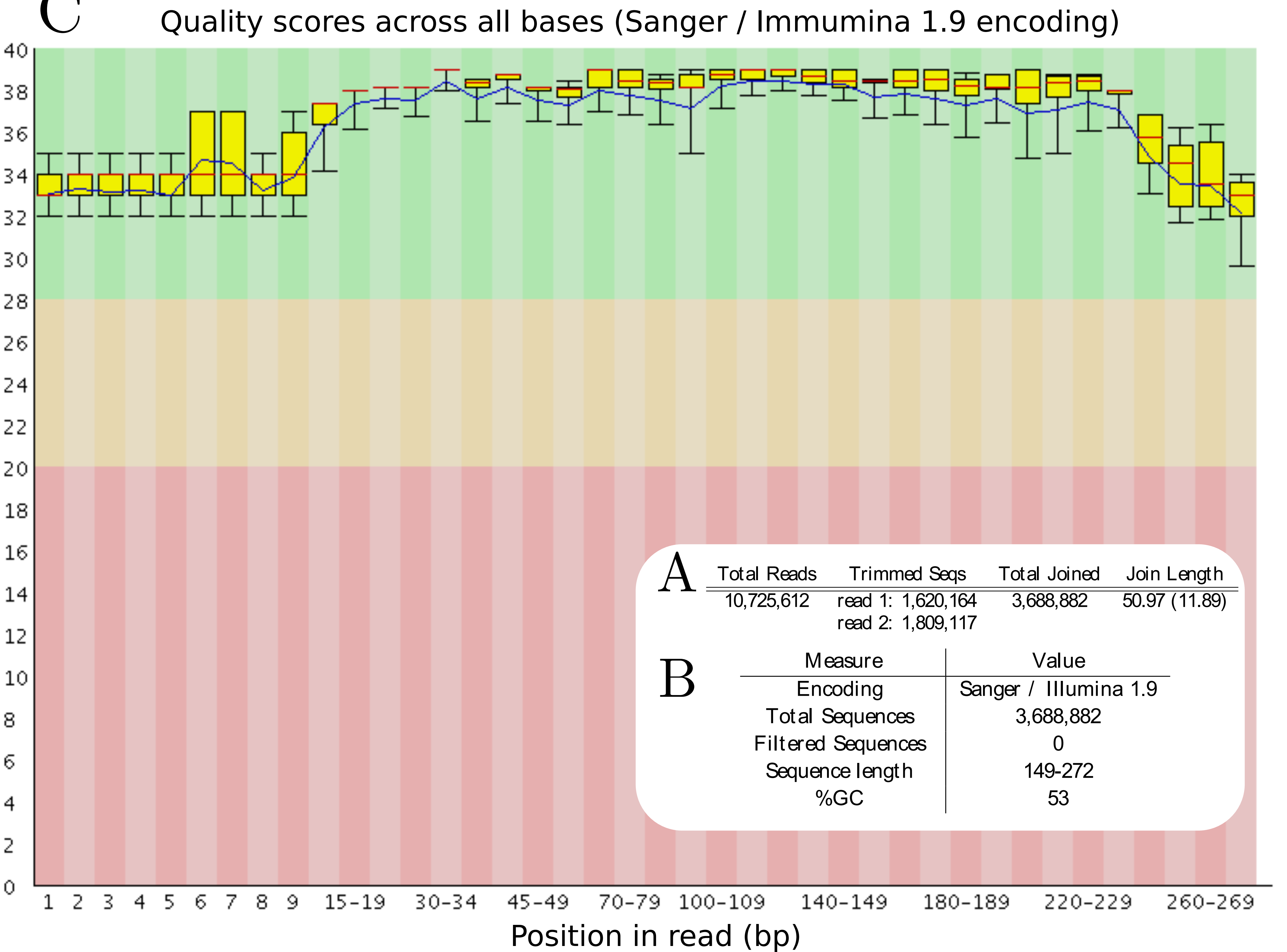

A

Total Reads	Trimmed Seqs	Total Joined	Join Length
10,725,612	read 1: 1,620,164<br>read 2: 1,809,117	3,688,882	50.97 (11.89)

B

Measure	Value
Encoding	Sanger / Illumina 1.9
Total Sequences	3,688,882
Filtered Sequences	0
Sequence length	149-272
%GC	53
