# Supplementary material for: Antibiotic perturbation of the murine gut microbiome enhances the adiposity, insulin resistance, and liver disease associated with high-fat diet
Source: Genome Med. 2016 Apr 27;8:48. doi: 10.1186/s13073-016-0297-9 (PMC4847194; doi:10.1186/s13073-016-0297-9)
Supplement: Additional file 3: Table S1. — Adonis tests of repeated measures. (DOCX 20 kb) [file 13073_2016_297_MOESM3_ESM.docx]

## Supplemental Table 1. Adonis tests of repeated measures.

### A. UniFrac distances for within subjects treatment effects

|  | Df | SumsOfSqs | MeanSqs | F Model | R^2^ | Pr(>F) |
| --- | --- | --- | --- | --- | --- | --- |
| Diet | 1 | 6.40 | 6.40 | 36.47 | 0.09 | 1.0000 |
| Treatment | 1 | 4.32 | 4.32 | 24.65 | 0.06 | **<0.0005** |
| Cage | 9 | 10.10 | 1.12 | 6.39 | 0.14 | 1.0000 |
| Diet:Treatment | 1 | 1.16 | 1.16 | 6.59 | 0.02 | **<0.0005** |
| Residuals | 290 | 50.88 | 0.18 |  | 0.70 |  |
| Total | 302 | 72.85 |  |  | 1.00 |  |

### B. UniFrac distances for within subjects disease effects

|  | Df | SumsOfSqs | MeanSqs | F Model | R^2^ | Pr(>F) |
| --- | --- | --- | --- | --- | --- | --- |
| Diet | 1 | 6.40 | 6.40 | 36.07 | 0.09 | **<0.0005** |
| Disease | 1 | 2.03 | 2.03 | 11.42 | 0.03 | **<0.0005** |
| Cage | 10 | 12.53 | 1.25 | 7.07 | 0.17 | 1.0000 |
| Diet:Disease | 1 | 0.62 | 0.62 | 3.49 | 0.01 | **<0.0005** |
| Residuals | 289 | 51.27 | 0.18 |  | 0.70 |  |
| Total | 302 | 72.85 |  |  | 1.00 |  |

We assessed the significance of UniFrac distances between Treatment (**A**) or Disease (**B**) microbiota using the Adonis function in the vegan package. To account for the multilevel design, we included a Diet:Treatment or Diet:Disease interaction term as well as cage effects for fitting the linear model. To reduce artificial inflation of the pseudo-F ratio, each of the 2000 independent sample permutations were restricted to within-subject and to within-Diet strata for constructing the empirical p-value.
