# Supplementary material for: Antibiotic perturbation of the murine gut microbiome enhances the adiposity, insulin resistance, and liver disease associated with high-fat diet
Source: Genome Med. 2016 Apr 27;8:48. doi: 10.1186/s13073-016-0297-9 (PMC4847194; doi:10.1186/s13073-016-0297-9)
Supplement: Additional file 8: Table S2. — Significant Taxa-host associations as determined from sparse PLS regression. p values are based on bootstrap evaluation of the PLS permutation test compared to a permutation-based null model on the variable support selected by sparse PLS. (PDF 43 kb) [file 13073_2016_297_MOESM8_ESM.pdf]

Significant Taxa-host associations as determined from sPLS regression

	TaxID	Rank2	Taxa	NAFLD	BMC	DMI	Fat	Lean	Weight	Weight+1
1	592160	p__Firmicutes	g__Lactobacillus	-	-	<5.000e-04 (-)	<5.000e-04 (-)	-	<5.000e-04 (-)	1.001e-03 (-)
2	1107027	p__Firmicutes	g__Lactobacillus	-	<5.000e-04 (-)	2.001e-03 (-)	-	<5.000e-04 (-)	7.004e-03 (-)	-
3	OTU1	p__Firmicutes	g__Lactobacillus	-	<5.000e-04 (-)	4.002e-03 (-)	-	<5.000e-04 (-)	3.002e-03 (-)	-
4	716006	p__Firmicutes	g__Lactococcus	-	<5.000e-04 (+)	<5.000e-04 (+)	<5.000e-04 (+)	<5.000e-04 (+)	<5.000e-04 (+)	<5.000e-04 (+)
5	324882	p__Firmicutes	g__Turicibacter	-	-	6.003e-03 (-)	7.004e-03 (-)	-	-	8.004e-03 (-)
6	OTU45	p__Firmicutes	g__Turicibacter	-	-	<5.000e-04 (-)	2.001e-03 (-)	-	-	-
7	835900	p__Bacteroidetes	g__Odoribacter	-	-	-	-	-	-	-
8	206817	p__Bacteroidetes	f__S24-7	-	-	-	-	-	-	1.001e-03 (+)
9	270984	p__Bacteroidetes	f__S24-7	-	-	5.003e-03 (-)	<5.000e-04 (-)	-	-	<5.000e-04 (-)
10	453896	p__Bacteroidetes	f__S24-7	-	-	-	2.001e-03 (-)	-	-	9.005e-03 (-)
11	184517	p__Bacteroidetes	f__S24-7	-	1.001e-03 (+)	-	-	1.001e-03 (+)	-	-
12	OTU4131	p__Bacteroidetes	f__S24-7	-	1.001e-03 (+)	-	-	3.002e-03 (+)	-	-
13	332666	p__Bacteroidetes	f__S24-7	-	2.001e-03 (+)	-	-	-	-	-
14	353073	p__Bacteroidetes	f__S24-7	-	<5.000e-04 (-)	<5.000e-04 (-)	1.001e-03 (-)	<5.000e-04 (-)	<5.000e-04 (-)	<5.000e-04 (-)
15	421792	p__Bacteroidetes	f__S24-7	-	<5.000e-04 (+)	9.005e-03 (+)	6.003e-03 (+)	<5.000e-04 (+)	-	-
16	338258	p__Bacteroidetes	f__S24-7	-	<5.000e-04 (+)	<5.000e-04 (+)	<5.000e-04 (+)	<5.000e-04 (+)	<5.000e-04 (+)	<5.000e-04 (+)
17	322839	p__Firmicutes	o__Clostridiales	-	<5.000e-04 (+)	<5.000e-04 (+)	<5.000e-04 (+)	<5.000e-04 (+)	<5.000e-04 (+)	<5.000e-04 (+)
18	268733	p__Firmicutes	f__Clostridiaceae	-	-	-	-	-	-	-
19	OTU30	p__Firmicutes	f__Clostridiaceae	-	-	-	-	-	-	-
20	OTU12816	p__Firmicutes	f__Clostridiaceae	-	-	-	-	-	-	7.004e-03 (-)
21	555945	p__Firmicutes	f__Clostridiaceae	<5.000e-04 (-)	<5.000e-04 (+)	<5.000e-04 (+)	<5.000e-04 (+)	<5.000e-04 (+)	1.001e-03 (+)	<5.000e-04 (+)
22	425767	p__Firmicutes	g__Candidatus Arthromitus	-	1.001e-03 (-)	-	-	2.001e-03 (-)	9.005e-03 (-)	-
23	22668	p__Firmicutes	g__Candidatus Arthromitus	6.003e-03 (-)	<5.000e-04 (-)	<5.000e-04 (-)	<5.000e-04 (-)	<5.000e-04 (-)	<5.000e-04 (-)	<5.000e-04 (-)
24	376862	p__Firmicutes	g__Candidatus Arthromitus	-	<5.000e-04 (-)	3.002e-03 (-)	-	<5.000e-04 (-)	<5.000e-04 (-)	-
25	352559	p__Firmicutes	f__Lachnospiraceae	-	-	6.003e-03 (+)	<5.000e-04 (+)	-	-	<5.000e-04 (+)
26	338796	p__Firmicutes	f__Ruminococcaceae	-	-	4.002e-03 (+)	9.005e-03 (+)	-	<5.000e-04 (+)	-
27	344101	p__Firmicutes	f__Ruminococcaceae	-	2.001e-03 (-)	4.002e-03 (-)	7.004e-03 (-)	<5.000e-04 (-)	-	<5.000e-04 (-)
28	230759	p__Firmicutes	g__Allobaculum	<5.000e-04 (-)	<5.000e-04 (+)	1.001e-03 (+)	9.005e-03 (+)	<5.000e-04 (+)	-	-
29	228310	p__Tenericutes	g__Anaeroplasma	-	<5.000e-04 (-)	<5.000e-04 (-)	<5.000e-04 (-)	<5.000e-04 (-)	<5.000e-04 (-)	<5.000e-04 (-)
